# Conditional deletion of human STN1 leads to telomere dysfunction, genome instability and proliferation defects

**DOI:** 10.1242/jcs.264269

**Published:** 2026-05-18

**Authors:** Jaclyn S. Holbrooks, Colin A. Loveless, Kennedy A. Vogler, S. Donte’ Reed, Grayson H. Duvall, Carlan V. Romney, Stephanie M. Ackerson, Madison B. Kircher, Jason A. Stewart

**Affiliations:** ^1^ Department of Biological Sciences, Western Kentucky University, Bowling Green, KY, 42101, USA; ^2^ Department of Biological Sciences, University of South Carolina, Columbia, SC 29208, USA

**Keywords:** STN1, CST, Telomere, Genome instability

## Abstract

CTC1–STN1–TEN1 (CST) is a heterotrimeric, RPA-like complex that binds single-stranded DNA, stimulates DNA polymerase α-primase, and functions in several genome maintenance pathways, including telomere maintenance and DNA replication and repair. During telomere replication, CST prevents telomerase from overextending the G-rich single-stranded DNA overhang (G-overhang) and promotes fill-in of the C-rich strand by stimulating DNA polymerase α-primase. Previous work characterized the effects of CST loss by deleting CTC1 or TEN1. Interestingly, CTC1 knockout (KO) caused severe proliferation defects and telomeric damage signaling, whereas these phenotypes were absent following TEN1 KO. Molecular analysis revealed that, while loss of CTC1 or TEN1 leads to defective C-strand fill-in, only CTC1 KO exhibited excessive G-overhang lengthening. Here, we characterized conditional STN1 KO cells and determined that STN1 KO leads to proliferation defects, telomeric damage signaling, and genome instability in the form of anaphase bridges and micronuclei. Our findings indicate that STN1 KO closely resembles CTC1 versus TEN1 KO and leads to increased genome instability.

## Teaser

**Summary:** The STN1 subunit of the single-stranded DNA-binding protein CST prevents telomeric damage signaling, genome instability and proliferation defects.

## INTRODUCTION

Human CTC1–STN1–TEN1 (CST) is a single-stranded DNA (ssDNA)-binding protein complex that functions in various genome maintenance pathways, including telomere length regulation, DNA replication and DNA repair (Lim and Cech, 2021; Lyu et al., 2021b; Olson and Wuttke, 2024; Stewart et al., 2018). CST consists of numerous oligonucleotide-oligosaccharide binding folds (OB-folds), which allow it to bind to ssDNA (Lim et al., 2020). It has conservation with the well-characterized ssDNA binding protein RPA (Barbour and Wuttke, 2023; Price et al., 2010). Like RPA, the use of multiple OB-folds allows CST to bind to DNA in a length-dependent manner and to different DNA configurations (Bhattacharjee et al., 2016). When all OB-folds are engaged, it binds in the low to sub nanomolar range (Bhattacharjee et al., 2016; Chen et al., 2012). However, unlike RPA, CST has some sequence preference for G-rich DNA (Hom and Wuttke, 2017).

A primary interacting partner of CST is DNA polymerase α (pol α)-primase, an interaction conserved from yeast to humans (Giraud-Panis et al., 2010; Price et al., 2010). CTC1 and STN1 were originally discovered in a screen for factors that co-purified with pol α-primase (Goulian et al., 1990). Originally named AAF (α-accessory factor), this complex was shown to stimulate pol α-primase activity (Goulian and Heard, 1990). Later work found that human OBFC1, which was later found to be one of the subunits of AAF, was a homolog of *Saccharomyces cerevisiae* Stn1, based on sequence similarity (Miyake et al., 2009). CTC1 and TEN1 were then identified as STN1-interacting partners by mass spectrometry. Independently, CTC1 was discovered in plants and mammals and found to interact with STN1 and TEN1 (Surovtseva et al., 2009). Since the subunits of AAF (CTC1 and STN1) were identified as components of CST, the complex was renamed to fit its homology to the budding yeast complex (Giraud-Panis et al., 2010; Price et al., 2010).

In *S. cerevisiae*, CST (Cdc13-Stn1-Ten1) plays an essential role in telomere maintenance (Giraud-Panis et al., 2010; Price et al., 2010). Mammalian and yeast CST both regulate telomere replication and interact with pol α-primase. CST stimulates the primase to polymerase transition as well as both primase and polymerase activities of pol α-primase (Casteel et al., 2009; Ganduri and Lue, 2017; Goulian and Heard, 1990; Goulian et al., 1990). This interaction is crucial for CST function in telomere replication as well as DNA repair (Cai and de Lange, 2023; Mirman et al., 2022).

In addition to pol α-primase, CST interacts with other proteins involved in DNA replication and repair, such as the MCM2-7 complex, cohesin, shieldin, RAD51, MRE11, RECQ4 (also known as RECQL4), OGG1 and DNA polymerase β, and functions in a variety of genome maintenance pathways, including DNA replication restart, double-strand break repair, origin licensing, base excision repair, protection of stalled replication forks, and maintenance of sister chromatid cohesion (Chastain et al., 2016; Hara et al., 2023; Lyu et al., 2021 a; Lyu et al., 2021 b; Mirman et al., 2018; Schuck et al., 2021; Stewart et al., 2012 b; Wang et al., 2019; Wysong et al., 2024). Previous and more recent work indicates that *S. cerevisiae* CST also functions in DNA replication (Calvo et al., 2019; Gasparayan et al., 2022; Gasparyan et al., 2009); however, its primary role is telomeric. These common but varied interactions suggest that CST acts as a general genome stability factor. The extent of its involvement in each pathway in humans is still under investigation; however, like in budding yeast, telomere maintenance is presumed to be its primary function.

Telomeres consist of repetitive DNA sequences (TTAGGG in humans) at the ends of chromosomes (Shay and Wright, 2019). In humans, these DNA regions can be several to tens of kilobases in length and consist of both a double-stranded and short ssDNA (100-300 nucleotide) region. The G-rich ssDNA region, located at chromosome termini, is known as the G-overhang. Telomeres are bound by a protection complex called shelterin, which blocks the ends from being recognized as DNA breaks and from nuclease degradation (de Lange, 2018).

Along with shelterin, CST functions in telomere maintenance and length regulation. Telomere replication occurs in three distinct steps (Bonnell et al., 2021; Stewart et al., 2012a). First, the telomere duplex region is replicated by the replisome with the help of additional accessory factors, including CST (Brenner and Nandakumar, 2022; Stewart et al., 2012b). In telomerase-positive cells (e.g. germline, stem and most cancer cells), the G-overhang is then extended through the reverse transcriptase activity of telomerase (Greider and Blackburn, 1985; Morin, 1989). Following initial extension, CST binds to inhibit telomerase from rebinding and overextending the G-overhang (Chen et al., 2012; Zaug et al., 2021). Most of the G-overhang is then converted to duplex DNA by pol α-primase, leaving a short overhang for formation of the protective telomeric-loop (Olson et al., 2022). This fill-in process, known as C-strand fill-in, is facilitated by CST-dependent stimulation of pol α-primase activity (Takai et al., 2024; Wang et al., 2012; Zaug et al., 2022). Recent work also demonstrated that the shelterin subunit POT1, which protects the G-overhang, recruits CST to regulate telomerase inhibition and C-strand fill-in (Cai et al., 2024).

To better understand the cellular roles of human CST, cell lines that conditionally deleted either CTC1 or TEN1 were previously generated (Feng et al., 2018; Feng et al., 2017). Conditional knockout (KO) of CTC1 results in significant growth defects and the accumulation of G2 cells starting around 10 days after CTC1 deletion (Ackerson et al., 2020; Feng et al., 2017). Molecular analysis of these cells demonstrated a severe defect in telomere length regulation, with G-overhangs reaching kilobases in length and telomeric damage signaling (i.e. RPA and γH2AX localization). Additional analysis revealed that the elongated G-overhangs were caused by the inability to inhibit telomerase and stimulate C-strand fill-in. Once overextended, there is insufficient POT1 to bind and protect the G-overhang. Typically, POT1 protects G-overhangs from repair, but due to the insufficient level of POT1, the now exposed ssDNA is bound by RPA, which leads to damage signaling mediated by the DNA damage response kinase ATR (Denchi and de Lange, 2007; Glousker et al., 2020). In contrast to CTC1 KO, conditional TEN1 KO exhibited no significant growth defect or telomeric damage signaling (Feng et al., 2018). Instead, there was a minor increase in G-overhang length, similar to what was previously observed with shRNA knockdown of each CST subunit (Kasbek et al., 2013; Miyake et al., 2009; Surovtseva et al., 2009; Wang et al., 2012). Molecular characterization of the KO cells showed that CTC1-STN1 can inhibit telomerase without TEN1, but TEN1 is required for the stimulation of pol α-primase to complete C-strand fill-in (Feng et al., 2018).

Unlike CTC1 and TEN1, a STN1 KO cell line has not yet been characterized. The goal of this study was to characterize conditional STN1 KO cells and compare whether the phenotype resembles CTC1 or TEN1 KO, or an intermediate phenotype. We determined that deletion of STN1 closely resembles deletion of CTC1, causing proliferation defects, telomeric RPA binding and telomeric damage signaling. Moreover, we determined that STN1 KO increased general genome instability, in the form of anaphase bridges and micronuclei. A recent study found that interaction between CTC1 and STN1 prevents CTC1 degradation, suggesting that the phenotypes observed following STN1 KO could be solely due to CTC1 degradation (Lan et al., 2025). However, we found that overexpression of CTC1 did not rescue the phenotypes caused by STN1 KO, indicating that STN1 functions directly in telomere maintenance and genome protection. These findings suggest that STN1 is necessary to prevent excessive G-overhang extension, genome instability and growth inhibition.

## RESULTS

### STN1 deletion leads to decreased proliferation and G2 arrest

**Fig. 1.**
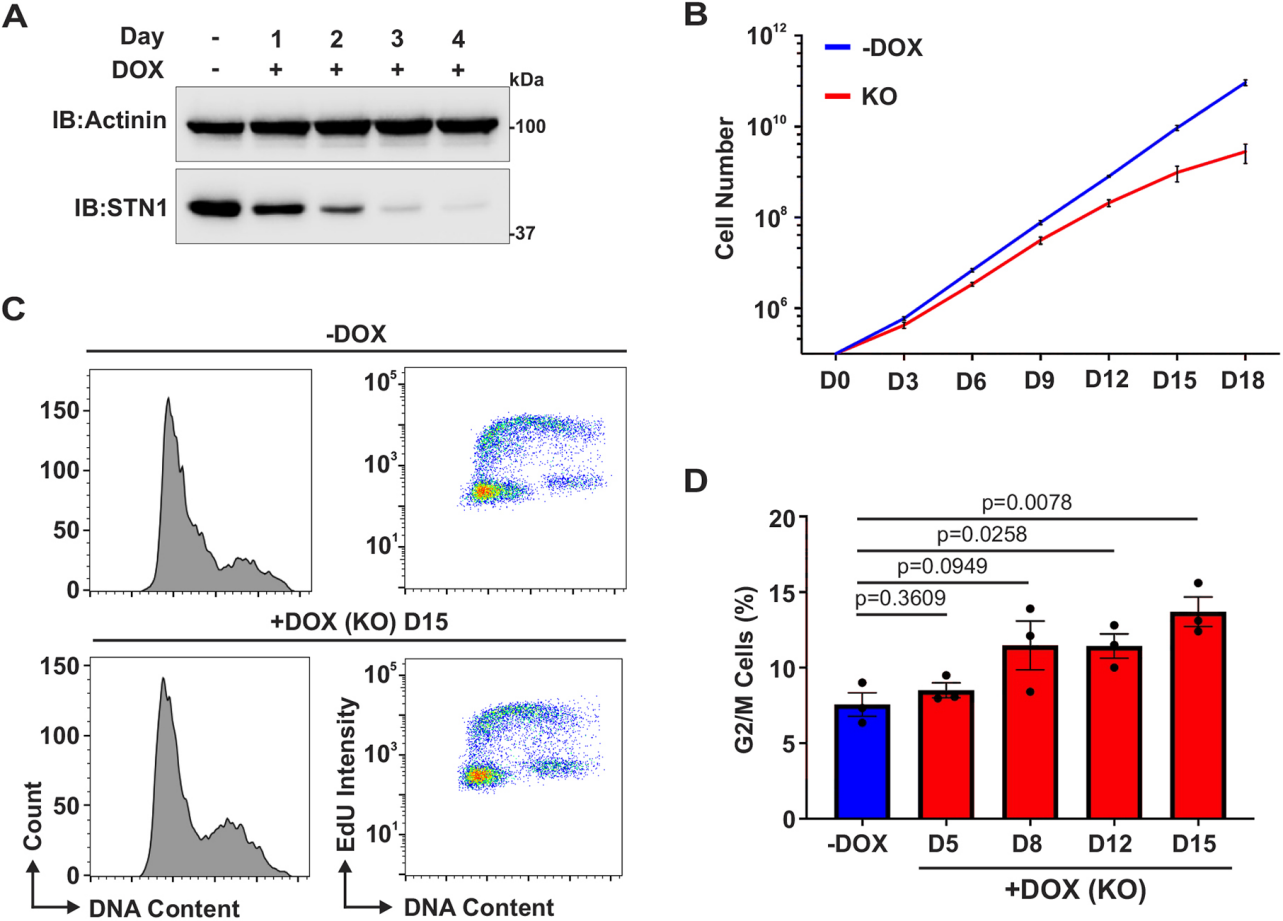
**STN1 KO leads to defects in cell proliferation.** (A) Western blot of STN1 levels in HeLa iCas9 sgSTN1 cells, as indicated. Actinin serves as a loading control. (B) Growth curve analysis of STN1 KO and control cells. *n*=3 independent, biological replicates. (C,D) Flow cytometry analysis of STN1 KO cells. (C) Representative histograms of STN1 KO and control (−DOX) cells. Left: Histograms of DNA content versus cell count. Right: DNA content versus EdU intensity. (D) Percentage of G2/M cells over time in STN1 KO cells. *n*=3 independent, biological replicates. Average values in the graphs indicate the mean and error bars denote s.e.m. *P*-values were calculated by a two-tailed, unpaired *t*-test. D, day after DOX addition; DOX, doxycycline.

To assess the phenotypes associated with the complete loss of STN1, we generated an inducible STN1 KO cell line. Single-guide RNAs to STN1 (sgSTN1) were generated, cloned into a retroviral vector, and transduced into previously characterized HeLa iCas9 cells, which contain Cas9 under an inducible doxycycline (DOX) promoter (McKinley, 2018; McKinley and Cheeseman, 2017). DOX was then added, and individual clones were screened for loss of STN1. One of the clones that displayed efficient gene disruption, which had little to no detectable STN1 after 4 days, was then selected for further characterization (Fig. 1A; Fig. S1A).

Growth curve analysis demonstrated our STN1 KO cells had a significant growth defect starting around day 9 after gene disruption (Fig. 1B). The timing of this growth defect is comparable to that observed in CTC1 KO cells (Feng et al., 2017). Extension of the growth curve to day 30 showed that growth was restored after ∼21 days (Fig. S1B). Western blot analysis of STN1 from day 5 to day 25 showed little to no detectable STN1 until around day 15, after which STN1 levels began to steadily increase (Fig. S1C). These results suggest that STN1 is not deleted in a small subset of the sgSTN1 cells following DOX treatment, and that these cells eventually outgrow the KO cells to become the dominant population. This finding is not unexpected in a conditional CRISPR-Cas9 KO cell line, where there could be incomplete editing and/or breaks that are repaired in-frame in a small subset of cells. The absence of STN1 gene disruption could also arise from a lack of Cas9 expression or the absence of sgSTN1. Because of the outgrowth of cells expressing STN1 starting around day 18 and rescue of cell proliferation around day 21, we confined our studies to days 5-15 when there is little to no detectable STN1 and growth is significantly decreased.

Previously, we determined that following CTC1 KO, there was an accumulation of G2-phase cells (Ackerson et al., 2020). Therefore, we performed cell cycle analysis between days 5 and 15 (Fig. 1C,D; Fig. S1D). This analysis showed an increase in G2/M cells starting around day 8. To distinguish between G2 and M phase, the mitotic index, as measured by phosphorylated histone H3 S10 (pH3 S10), was then determined (Fig. S1E). The percentage of mitotic cells was unaffected in the STN1 KO compared to control cells. These results indicate that deletion of STN1 leads to the accumulation of G2 cells, similar to CTC1 KO (Ackerson et al., 2020). Combined, these results indicate that STN1 KO leads to defects in cell proliferation and cell cycle progression.

### Loss of STN1 results in defective telomere length regulation and telomeric RPA

**Fig. 2.**
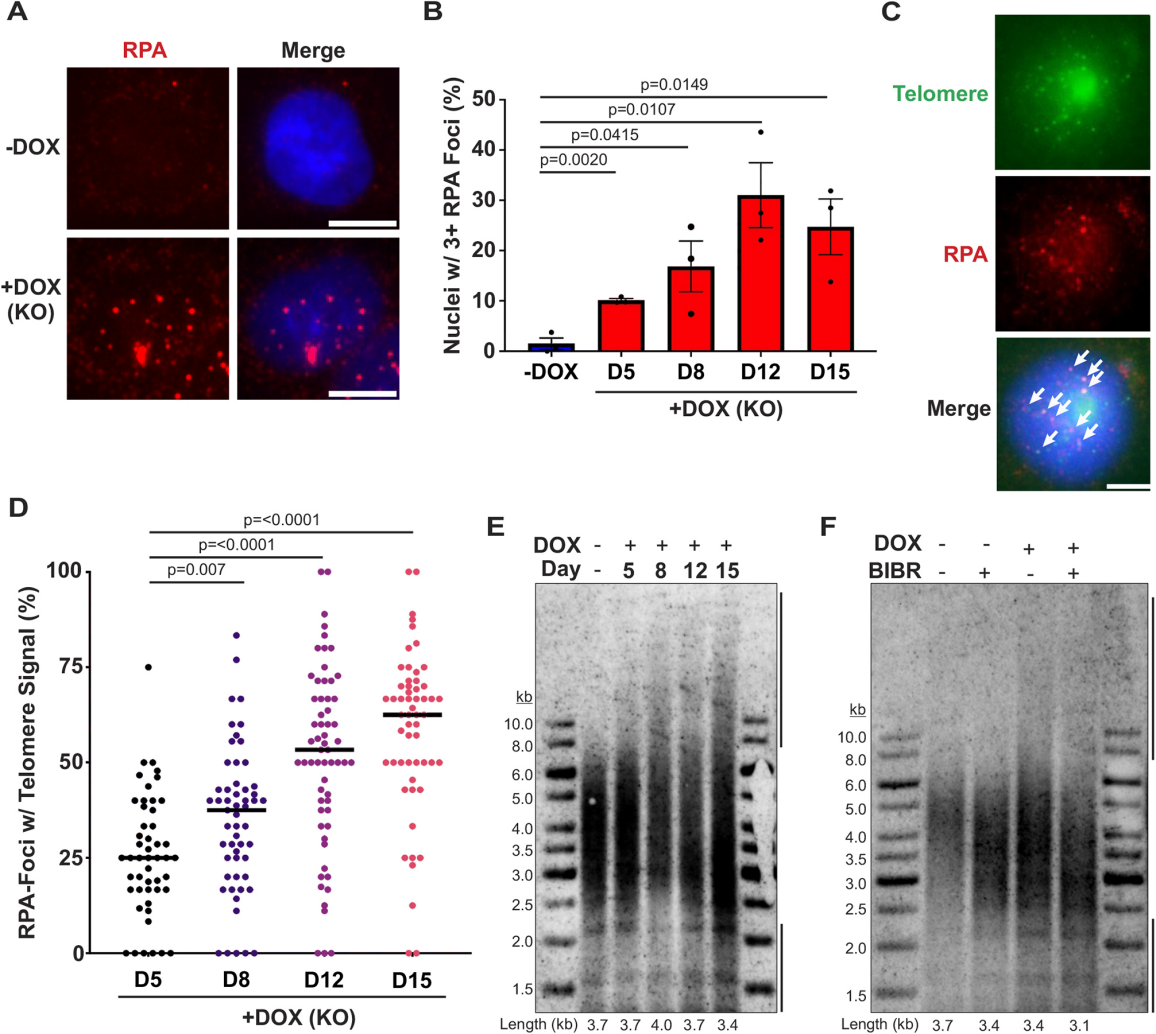
**STN1 KO leads to telomeric RPA binding.** (A) Representative images of RPA foci. STN1 KO image from day 15 after DOX addition. Blue, DAPI staining. Scale bars: 10 µm. (B) Analysis of RPA foci in STN1 KO and control (−DOX) cells. Percentage of nuclei displaying three or more RPA foci. *n*=3 independent, biological replicates. (C) Representative images of RPA localization to telomeres in STN1 KO cells on day 15 after DOX addition. The arrows denote colocalizations. Blue, DAPI staining. Scale bar: 5 µm. (D) Dot plot of the percentage of RPA-foci colocalizing with telomere signal. Each dot represents an individual nucleus. *n*=3 independent, biological replicates. (E,F) Southern blot analysis of the terminal restriction fragments from day 5 to 15 after DOX addition (E) and with BIBR1532 (telomerase inhibitor) or DMSO treatment (F). Lines on the right side of the gel denote telomere elongation and telomere shortening in the STN1 KO cells. Mean telomere length is indicated below the gel. Average values in the graphs indicate the mean and error bars denote s.e.m. *P*-values were calculated by a two-tailed, unpaired *t*-test for B and Mann–Whitney *U-*test for D. D, day after DOX addition; DOX, doxycycline.

The accumulation of G2 cells and proliferation defect did not occur until around 9 days after DOX addition, suggesting that, like CTC1 KO, these phenotypes may arise from RPA binding and ATR-mediated telomeric damage signaling due to G-overhang overextension (Ackerson et al., 2020; Feng et al., 2017). To confirm this, we measured the number of RPA foci in STN1 KO cells between days 5 and 15 by immunofluorescence (IF) (Fig. 2A,B). We found that the number of cells with RPA foci increased significantly over time. To determine whether the RPA foci were telomeric, IF combined with fluorescence *in situ* hybridization (IF-FISH) analysis was then performed with an antibody to RPA and a telomere probe (Fig. 2C,D). From day 5 to day 15, there was a gradual increase in telomeric RPA foci, which increased from around 25% at day 5 to 60% at day 15 (Fig. 2D). Similar results were previously observed at day 15 with CTC1 KO, which showed ∼80% RPA localization at telomeres (Ackerson et al., 2020). These results suggest that the low levels of RPA at day 5 are mostly non-telomeric, but at later time points RPA binds to G-overhangs that are overextended by telomerase in the absence of STN1.

To confirm that telomere maintenance was dysregulated following STN1 deletion, we next performed terminal restriction fragment Southern blot analysis (Fig. 2E). Telomere length became more heterogeneous over time in the STN1 KO cells, showing both an increase and a decrease compared to the −DOX controls. This pattern resembles a shift in telomere signal in CTC1 KO cells from day 10 to day 14 after conditional deletion (Feng et al., 2017). These results suggest that, like CTC1 KO, loss of STN1 leads to G-overhang elongation due to the absence of telomerase inhibition as well as a shortening of the duplex DNA due to defects in C-strand fill.

To verify that this increase in telomere length was telomerase dependent, cells were treated with the telomerase inhibitor BIBR1532. This decreased the long telomere products at the top of the blot in the STN1 KO cells while the shorter telomere products remained unchanged (Fig. 2F), similar to what was observed in CTC1 KO cells (Feng et al., 2018). Furthermore, BIBR1532 treatment decreased the number of RPA foci in STN1 KO cells (Fig. S2). Our findings demonstrate that the increased telomere length is telomerase dependent and leads to RPA binding in STN1 KO cells due to G-overhang elongation.

### STN1 deletion leads to telomeric DNA damage signaling and ATR activation

**Fig. 3.**
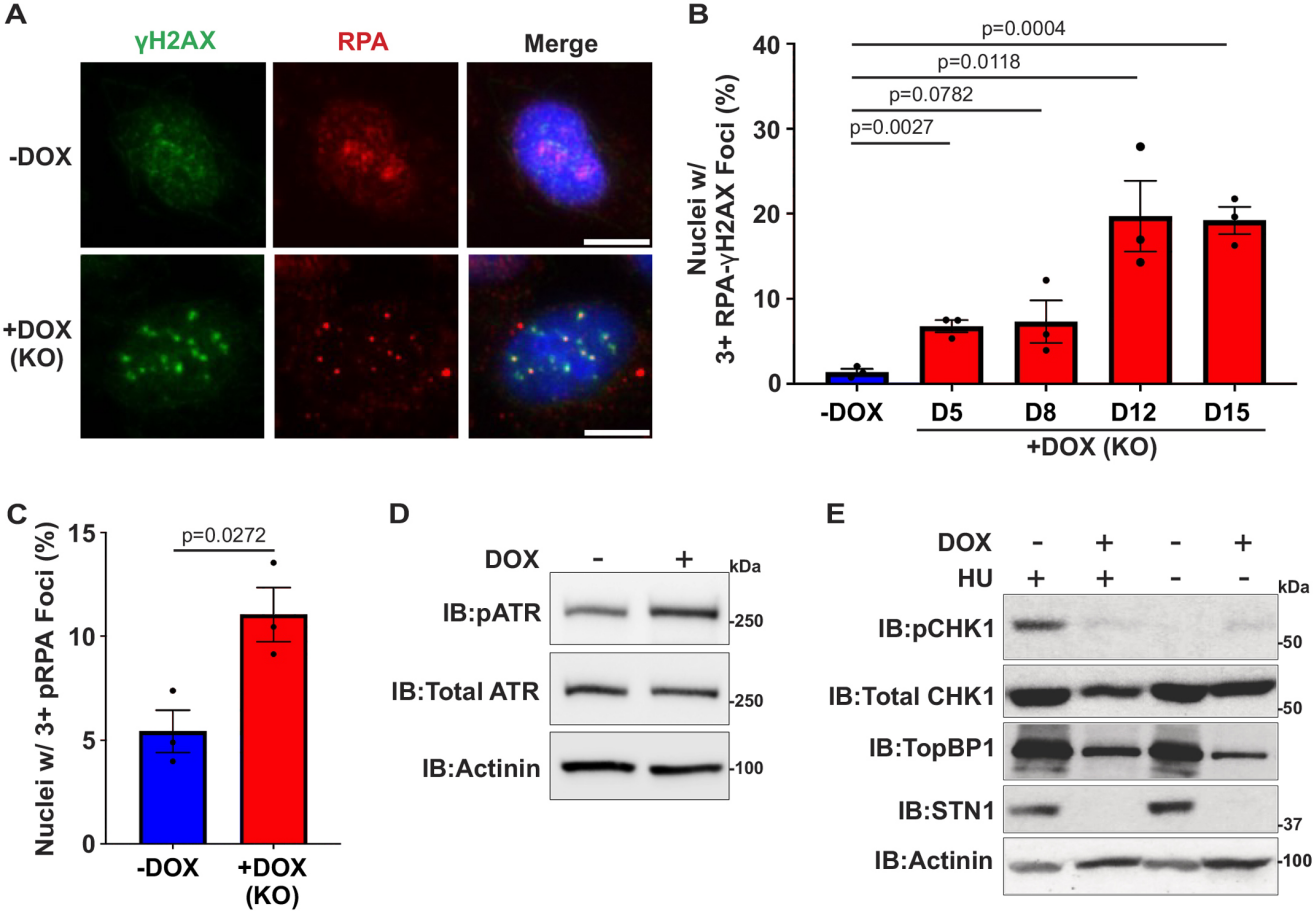
**STN1 KO activates DNA damage signaling but not CHK1 phosphorylation.** (A) Representative images of RPA-γH2AX foci. STN1 KO image is from day 12 after DOX addition. Blue, DAPI staining. Scale bars: 10 µm. (B) Analysis of foci in STN1 KO and control (−DOX) cells. Percentage of nuclei displaying three or more RPA-γH2AX foci. *n*=3 independent, biological replicates. (C) Analysis of phosphorylated RPA32 S33 (pRPA) foci in STN1 KO and control cells. Percentage of nuclei displaying three or more pRPA foci. *n*=3 independent, biological replicates. (D) Western blot of phosphorylated ATR T1989 (pATR) and total ATR levels on day 15. Actinin serves as a loading control. (E) Western blot of CHK1 phosphorylation and TopBP1 levels on day 18, as indicated. Cells were treated with hydroxyurea (HU) for 2 h prior to collection, as indicated. Actinin serves as a loading control. Average values in the graphs indicate the mean and error bars denote s.e.m. *P*-values were calculated by a two-tailed, unpaired *t*-test. D, day after DOX addition; DOX, doxycycline.

Next, we determined whether RPA-binding resulted in telomeric damage signaling and ATR activation (Ackerson et al., 2020; Feng et al., 2017). Colocalization between RPA and γH2AX was measured in STN1 KO cells between day 5 and day 15 (Fig. 3A,B). While there was a slight increase in cells with RPA-γH2AX foci on days 5 and 8, the levels more than doubled at days 12 and 15. These days correspond to the decreased proliferation and increased G2-phase cells observed after STN1 KO (Fig. 1; Fig. S1). Furthermore, we determined that the majority of these γH2AX foci were telomeric by IF-FISH (Fig. S3). Since γH2AX can be phosphorylated by several DNA damage response kinases, we measured the levels of phosphorylated RPA32 S33 (pRPA), which is an ATR-specific phosphorylation (Marechal and Zou, 2015). pRPA foci were significantly increased in the STN1 KO cells compared to controls (Fig. 3C). As further confirmation of ATR activation, western blot analysis was conducted to measure ATR phosphorylation at T1989 (pATR), a marker of ATR activation (Nam et al., 2011). pATR was increased ∼1.7-fold in the STN1 KO cells compared to the −DOX control (Fig. 3D).

Previous work found that despite ATR activation at telomeres in CTC1 KO cells, the downstream effector kinase CHK1 was not activated, and levels of the ATR activator TopBP1 were decreased (Ackerson et al., 2020). Moreover, phosphorylated CHK1 (pCHK1) was compromised following hydroxyurea-induced replication stress. Accordingly, we tested whether CHK1 phosphorylation was affected in STN1 KO cells and found that STN1 deletion leads to similar defects in global ATR-CHK1 signaling and decreased TopBP1 levels (Fig. 3E). Together, these results indicate that STN1 deletion leads to telomere damage signaling and ATR activation; however, this did not result in CHK1 activation, similar to CTC1 deletion.

### Add back of STN1 rescues cell growth and RPA foci in STN1 KO cells

**Fig. 4.**
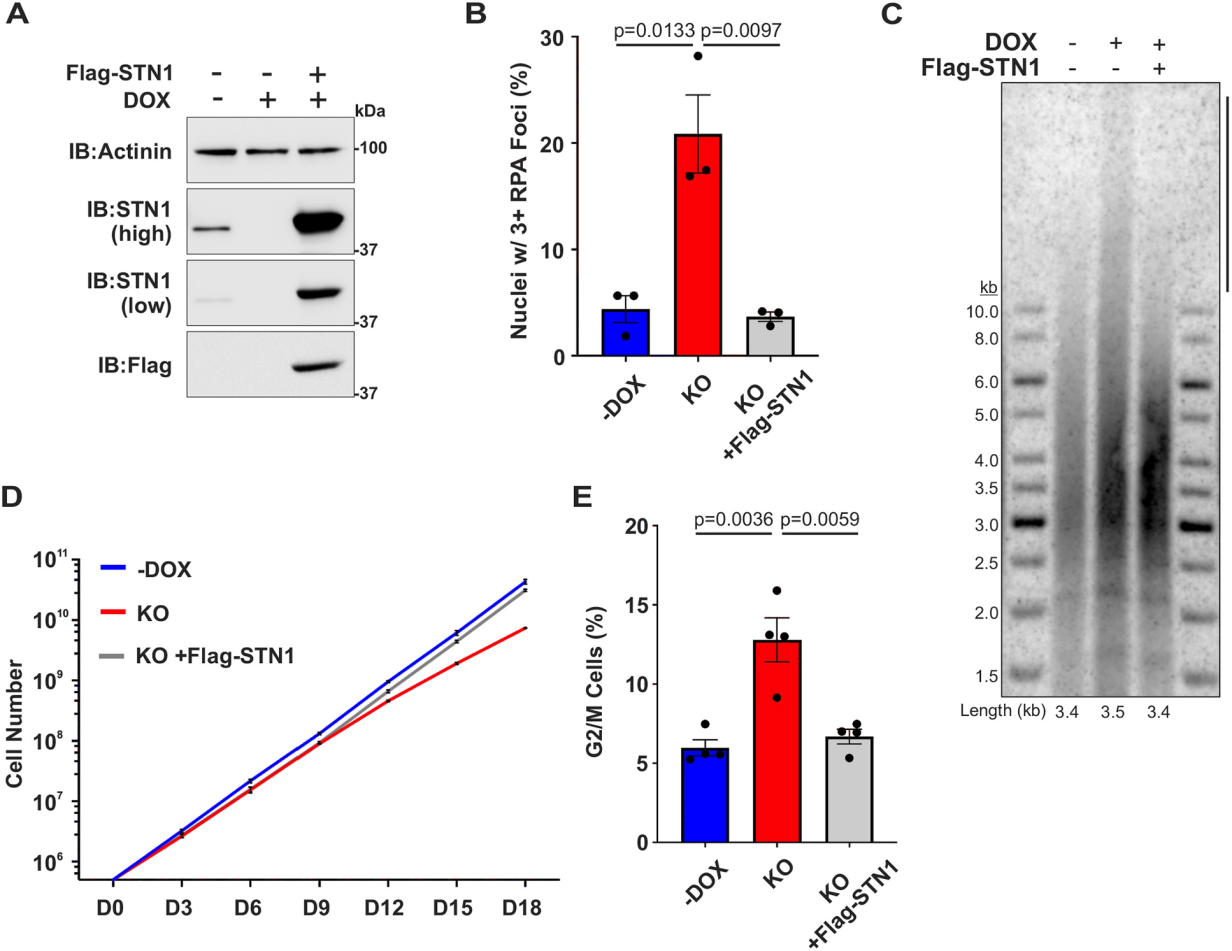
**Expression of Flag-STN1 rescues RPA foci and cell proliferation defects in STN1 KO cells.** (A) Western blot of STN1 and Flag-STN1 levels on day 15, as indicated. KO+Flag-STN1 denotes STN1 KO cells expressing Flag-STN1. Actinin serves as a loading control. High and low indicate longer and shorter exposures of the blot, respectively. (B) Percentage of nuclei displaying three or more RPA foci on day 15 after DOX addition. *n*=3 independent, biological replicates. (C) Southern blot analysis of the terminal restriction fragments, as indicated. Line on the right side of the gel denotes telomere elongation. Mean telomere length is indicated below the gel. (D) Growth curve analysis, as indicated. *n*=3 independent, biological replicates. (E) Percentage of G2/M cells determined by flow cytometry on day 15 after DOX addition. *n*=4 independent, biological replicates. Average values in the graphs indicate the mean and error bars denote s.e.m. *P*-values were calculated by a two-tailed, unpaired *t*-test. D, day after DOX addition; DOX, doxycycline.

To validate that the phenotypes observed were due to STN1 deletion and not off-target defects, we created a cell line expressing an sgRNA-resistant Flag-STN1 (KO+Flag-STN1). We confirmed the expression of Flag-STN1 by western blotting (Fig. 4A). The number of cells with RPA foci was then measured on day 15, which corresponds to severe growth inhibition and telomeric damage signaling (see Figs 1, 2). The expression of Flag-STN1 rescued the RPA foci (Fig. 4B). Similarly, telomere elongation was rescued in KO cells expressing Flag-STN1 (Fig. 4C). Growth curve and cell cycle analysis were then performed, and, again, expression of Flag-STN1 rescued both cell growth and the increase in the number of cells in G2 phase (Fig. 4D,E), indicating these phenotypes result from STN1 deletion and not potential off-target effects of the CRISPR-Cas9 system.

### STN1 deletion causes general genome instability phenotypes

**Fig. 5.**
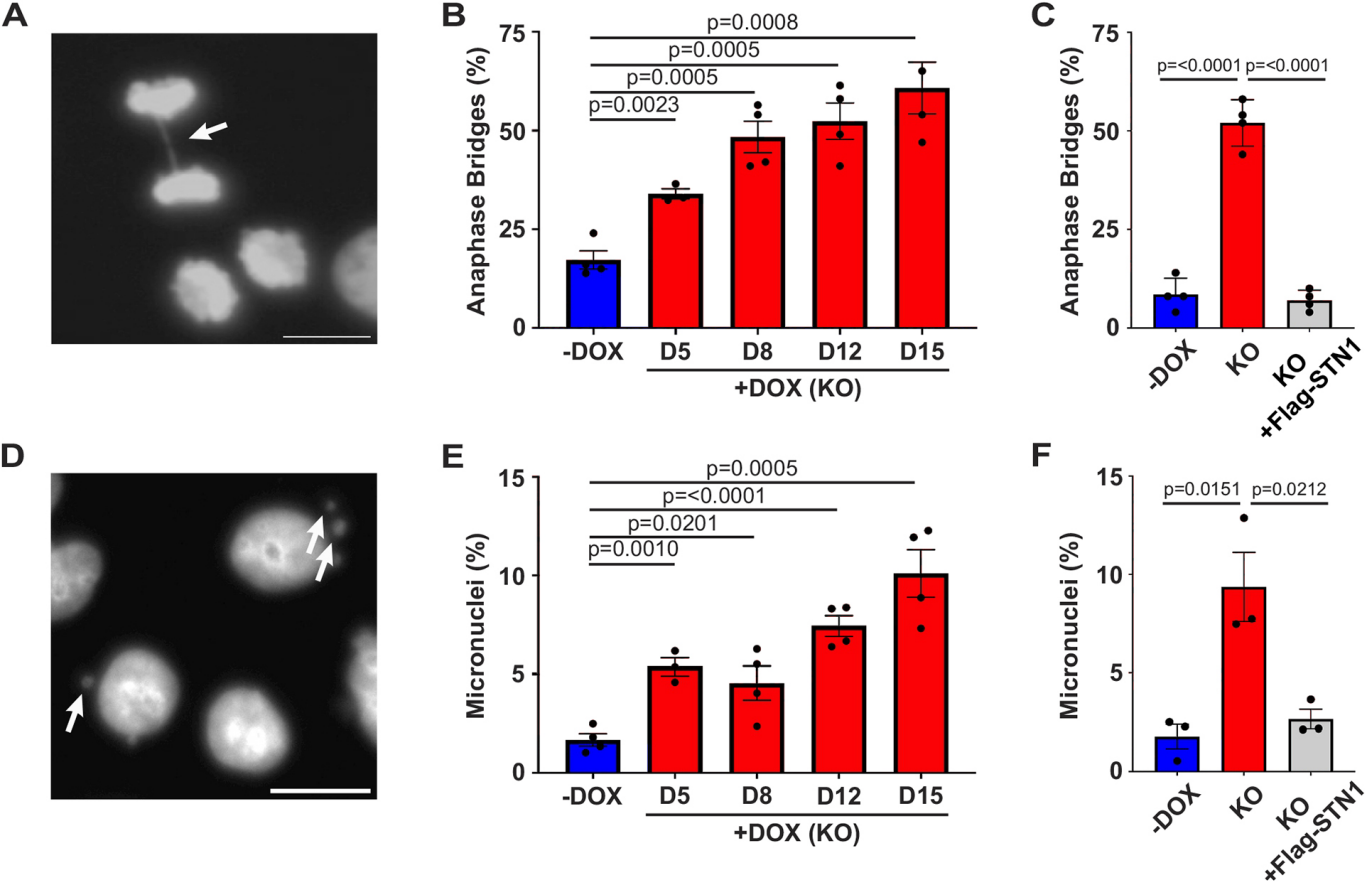
**STN1 KO increases general genome instability.** (A) Representative image of an anaphase bridge (indicated by the arrow) in STN1 KO cells. Scale bar: 20 µm. (B,C) Percentage of anaphase bridges in STN1 KO and control (−DOX) cells between days 5 and 15 (B) or with expression of exogenous Flag-STN1 on day 15 (C). *n*≥3 independent, biological replicates. (D) Representative image of micronuclei (indicated by the arrows) in the STN1 KO cells. Scale bar: 20 µm. (E,F) Percentage of cells with micronuclei in STN1 KO and control cells between days 5 and 15 (E) or with expression of exogenous Flag-STN1 on day 15 (F). *n*≥3 independent, biological replicates. Average values in the graphs indicate the mean and error bars denote s.e.m. *P*-values were calculated by a two-tailed, unpaired *t*-test. D, day after DOX addition; DOX, doxycycline.

We and others previously determined that STN1 knockdown with shRNA or siRNA increases general genome instability in the form of anaphase bridges and micronuclei (Bhattacharjee et al., 2016; Lyu et al., 2021a; Stewart et al., 2012b; Surovtseva et al., 2009). To determine whether STN1 KO also displayed these phenotypes, we measured anaphase bridges and micronuclei between days 5 and 15 (Fig. 5). There was a significant increase in both phenotypes starting at day 5, with the levels continuing to increase through day 15 (Fig. 5B,E). Expression of Flag-STN1 rescued the increases in both anaphase bridges and micronuclei on day 15 (Fig. 5C,F). We previously showed that STN1 deletion increases the number of G2-phase cells without affecting the number of mitotic cells (Fig. 1D; Fig. S1E). As progression to mitosis would be required for the formation of anaphase bridges and micronuclei, we tested whether cells with RPA foci progressed into mitosis by staining for both RPA and pH3 S10, as a marker of mitotic cells (Fig. S4). Mitotic cells with RPA foci were significantly increased in STN1 KO cells compared to the controls. These findings suggest that the presence of RPA foci is not necessarily sufficient to arrest cells in G2. This could be due to these cells moving more slowly through G2 but eventually entering mitosis, or that they need to accumulate enough RPA-bound telomeres to become arrested in G2. Overall, these findings indicate that STN1 KO results in genome instability.

### Overexpression of CTC1 does not rescue STN1 KO phenotypes

**Fig. 6.**
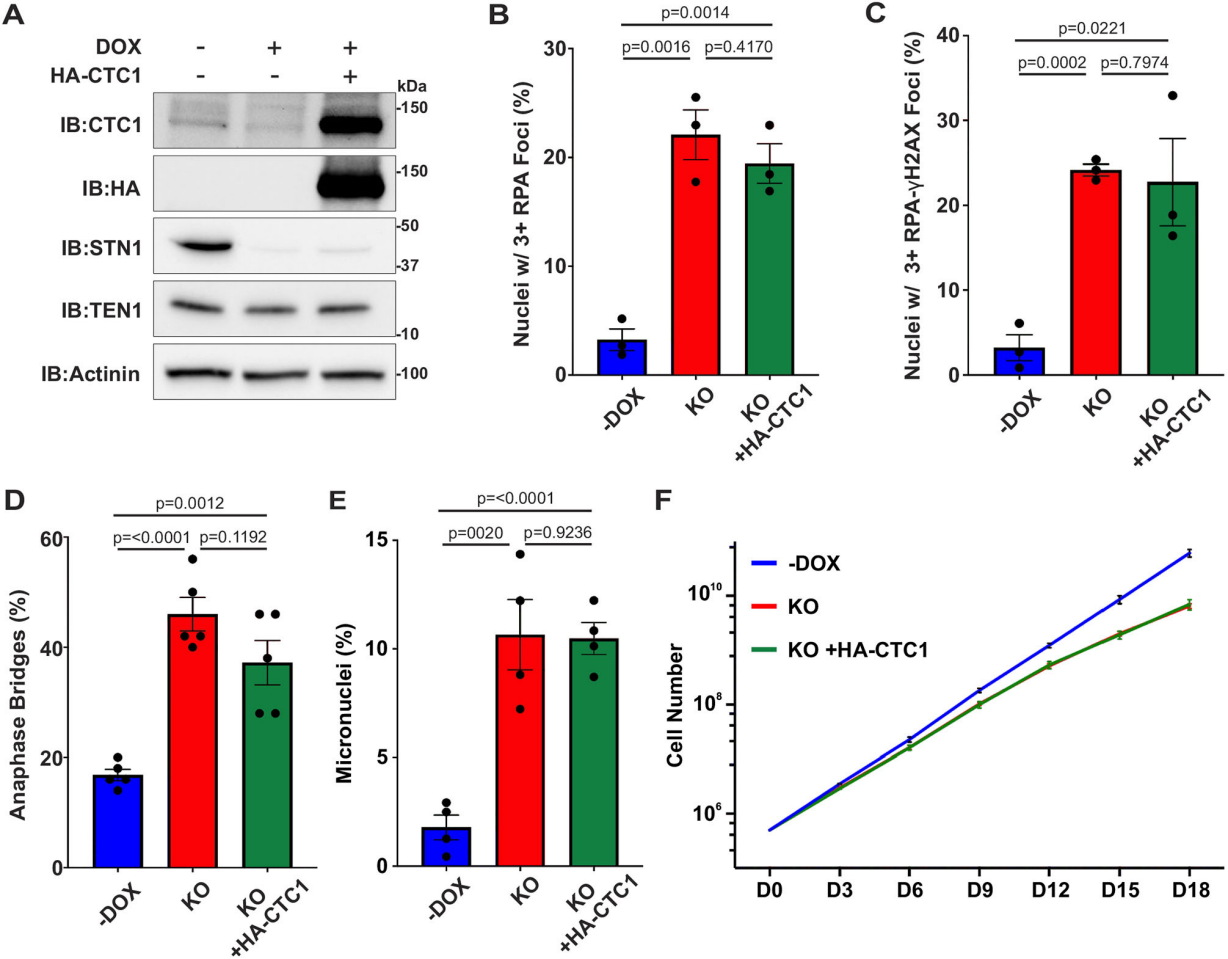
**CTC1 overexpression does not rescue STN1 KO phenotypes.** (A) Western blot of CTC1, STN1 and TEN1 expression on day 15, as indicated. Actinin serves as a loading control. (B,C) Percentage of nuclei displaying three or more RPA (B) or RPA-γH2AX (C) foci on day 15 after DOX addition. *n*=3 independent, biological replicates. (D,E) Percentage of cells with anaphase bridges (D) or micronuclei (E) on day 15. *n*≥3 independent, biological replicates. (F) Growth curve analysis, as indicated. *n*=3 independent, biological replicates. Average values in the graphs indicate the mean and error bars denote s.e.m. *P*-values were calculated by a two-tailed, unpaired *t*-test. D, day after DOX addition; DOX, doxycycline. KO+HA-CTC1 denotes STN1 KO cells expressing HA-CTC1.

A recent study found that STN1 is necessary to prevent CTC1 degradation by blocking the interaction between the ubiquitin ligase TRIM32 and CTC1 (Lan et al., 2025). When STN1 was depleted or CTC1-STN1 interaction was disrupted, CTC1 degradation increased. Therefore, we wondered whether the observed phenotypes in our STN1 KO cells could be attributable to CTC1 protection or a direct role in telomere maintenance. We hypothesized that STN1 is directly involved in telomere maintenance and genome stability, given its crucial role in CST ssDNA binding (Bhattacharjee et al., 2016; Feng et al., 2018). To test our hypothesis, we generated a cell line overexpressing an HA-tagged CTC1. As previously reported (Lan et al., 2025), we observed a significant decrease in CTC1 following STN1 deletion (Fig. 6A). However, CTC1 overexpression was able to overcome CTC1 degradation in the absence of STN1. We then measured telomere dysfunction, genome instability and cell proliferation in STN1 KO cells overexpressing CTC1 (Fig. 6B-F). There was little to no change in the number of RPA-γH2AX foci, micronuclei, or cell proliferation compared to the KO cells. While there was a minor decrease in RPA foci and anaphase bridges, these changes were not statistically significant. These results indicate that CTC1 overexpression was unable to rescue these phenotypes and that STN1 plays a direct role in telomere maintenance and genome stability.

## DISCUSSION

Previous work considered the effects of CTC1 or TEN1 KO in human cells (Ackerson et al., 2020; Feng et al., 2018; Feng et al., 2017). In this work, we studied the impact of conditional STN1 KO. Loss of STN1 resulted in severe growth defects, the accumulation of G2-phase cells, and telomeric damage signaling from excessive G-overhang elongation. These findings suggest that STN1 KO resembles that of CTC1 KO rather than TEN1 KO. Moreover, we show that STN1 KO leads to significant increases in anaphase bridges and micronuclei. Finally, we determined that STN1 plays a direct role in telomere maintenance and genome stability beyond its newly discovered role in shielding CTC1 from degradation (Lan et al., 2025).

The CTC1 and TEN1 KO studies were performed in HCT116 cell lines by inserting loxP sites and then inducing Cre nuclease nuclear localization to remove the floxed region (Feng et al., 2018; Feng et al., 2017). Efforts to create a similar cell line for STN1 were unsuccessful, so we used a previously characterized HeLa inducible Cas9 cell line to conditionally delete STN1, using CRISPR-Cas9 technology (Cong et al., 2013; Jinek et al., 2013). Even though the cell type and mode of gene disruption differ between the CTC1 and STN1 KO lines, the phenotypes closely mirror one another. Deletion of CTC1 or STN1 has a profound, but not immediate, impact on cell growth and cell cycle progression (Ackerson et al., 2020; Feng et al., 2017) (Fig. 1). In contrast, deletion of TEN1 has little impact on cell proliferation (Feng et al., 2018).

Biochemical reconstitution demonstrates how CST orchestrates telomere replication by limiting telomerase activity and then stimulating pol α-primase activity to complete C-strand fill-in (Takai et al., 2024; Zaug et al., 2022; Zaug et al., 2021). The contributions of each subunit to these essential processes are highlighted by these gene KO experiments (Ackerson et al., 2020; Feng et al., 2018; Feng et al., 2017) (this study). Loss of either CTC1 or STN1 leads to excessive, telomerase-dependent G-overhang lengthening, leading to telomeric RPA and γH2AX, whereas TEN1 KO leads to a modest ∼2-fold increase in G-overhang signal and the absence of telomeric damage signaling (Feng et al., 2018). Recent work by Lan et al. found that STN1 and the E3 ubiquitin ligase TRIM32 compete for binding to the ‘cleft’ motif in CTC1, and loss of STN1 binding results in CTC1 ubiquitylation and degradation (Lan et al., 2025). Therefore, STN1 KO might phenocopy CTC1 KO because CTC1 is lost due to the absence of STN1. Interestingly, overexpression of CTC1 was unable to rescue the phenotypes we measured in the STN1 KO cells, indicating that both CTC1 and STN1 are required for telomerase inhibition and to prevent telomeric damage signaling. This fits with previous data demonstrating that CTC1 only weakly binds to ssDNA in the absence of STN1 and that STN1 interacts directly with pol α-primase and POT1 (Bhattacharjee et al., 2016; Cai et al., 2024; Carvalho Borges et al., 2023; Feng et al., 2018; He et al., 2022).

Analysis of CTC1 KO cells demonstrated that the overextended G-overhang eventually depletes the available pools of POT1, leading to RPA binding, ATR localization and H2AX phosphorylation (Ackerson et al., 2020; Feng et al., 2017). Consistent with this model, cells derived from CTC1 KO mice showed G-overhang overextension, telomeric γH2AX foci and cell proliferation defects (Gu et al., 2012). It is interesting to note that, while some telomeric RPA foci occur at earlier time points, the growth defect in CTC1 and STN1 KO cells does not occur until later, around day 9 (Fig. 1) (Feng et al., 2017). In the STN1 KO, there is an ∼20% increase in telomeric RPA and a 2.7-fold increase in RPA-γH2AX foci from day 8 to day 12, which correlates with growth inhibition (Fig. 1B). An ∼20% increase in telomeric γH2AX from day 7 to day 12 was also reported following CTC1 KO (Feng et al., 2017). These results suggest that a certain level of ATR-mediated telomeric damage signaling is tolerated before inducing global cell cycle arrest. This is consistent with previous studies demonstrating that the recruitment of ATR-ATRIP and associated ATR activators to RPA-bound DNA – sufficient to induce global checkpoint activation – is length dependent (Bantele et al., 2019; Choi et al., 2010; Cortez et al., 2001; Saldivar et al., 2017; Zou and Elledge, 2003).

We therefore propose that the growth inhibition caused by G-overhang overextension requires the accumulation of sufficient telomeric RPA to activate a global, ATR-mediated checkpoint response, followed by senescence or apoptosis. Both senescence and apoptosis have been observed in CTC1 KO cells (Ackerson et al., 2020; Feng et al., 2017; Gu et al., 2012). This model highlights the essential roles of POT1 and CST in telomere protection, with POT1 preventing RPA association and POT1-recuited CST inhibiting telomerase to prevent G-overhang elongation, which can lead to POT1 exhaustion and subsequent RPA binding. Such a model would be consistent with the fact that cells with RPA-foci can progress into mitosis (Fig. S4). Additional work will be necessary to determine the timing of ATR-ATRIP recruitment to overextended G-overhang and how this then leads to checkpoint activation, particularly since the G2 arrest in CTC1 and STN1 KO cells is independent of CHK1 (Ackerson et al., 2020) (Fig. 3E), and how RPA-bound telomeres remain protected from DNA repair mechanisms since only a minor increase in chromosome fusions were observed CTC1 KO cells (Feng et al., 2017).

## MATERIALS AND METHODS

### Cell culture

HeLa iCas9 cells were maintained in DMEM supplemented with 10% fetal bovine serum and 1% penicillin/streptomycin and grown at 37°C with 5% CO_2_. Cell lines were regularly checked for contamination. HeLa iCas9 (inducible Cas9) cells were generously provided by Dr Iain Cheeseman from the Massachusetts Institute of Technology (McKinley and Cheeseman, 2017). Generation of the STN1 inducible KO cell line was previously described (Wysong et al., 2024). Cell lines expressing Flag-STN1 or HA-CTC1 were made by transducing the HeLa iCas9 sgSTN1 cells with retrovirus generated from a pMIT vector encoding sgRNA-resistant Flag-STN1 or HA-CTC1. Cells were then selected by flow cytometry for the expression of Thy1.1. To induce conditional gene KO of STN1, cells were incubated with 1 μg/ml DOX for 4-5 days. In Fig. 2F and Fig. S2, cells were treated with BIBR1532 (telomerase inhibitor), which was added fresh every 2-3 days when cells were passaged. The telomerase inhibitor BIBR1532 (Selleckchem, S1186), which was dissolved in dimethyl sulfoxide (DMSO), was added at a final concentration of 20 μM starting on day 0. DMSO control samples were treated with an equivalent amount of DMSO at each cell passage.

### Growth curve

Cells were plated at 5×10^5^ and allowed to grow for 3 days. They were then counted and re-plated at 5×10^5^. This was repeated until the indicated time point, and the total number of cells was extrapolated from the cell counts to generate the graphs.

### Flow cytometry

Cells were collected and samples prepared as previously described (Ackerson et al., 2020), except that they were incubated for 1 h with 5-ethynyl-2′-deoxyuridine (EdU) prior to collection. The samples were then run on a BD FACSMelody (10,000 cells/biological replicate) and analysis performed using FlowJo 10 software.

### Antibodies

Primary antibodies used were: OBFC1 (STN1) (Novus Biologicals, NBP2-01006), α-Actinin (Santa Cruz Biotechnology, sc17829), pH3 S10 (Cell Signaling Technology, 9706), RPA32 (Abcam, ab16850), γH2AX (Bethyl, A300-081A; Abcam, ab81299), pRPA32 S33 (Bethyl, A300-246A), pATR T1989 (Cell Signaling Technology, 30632), ATR (Cell Signaling Technology, 13934), pCHK1 S317 (Bethyl, A304-673A), CHK1 (Cell Signaling Technology, 2360S), TopBP1 (Bethyl, A300-111A), Flag (Cell Signaling Technology, 14793S), CTC1 (Feng et al., 2017), TEN1 (Aviva Systems Biology, ARO90551_P050) and HA (Cell Signaling Technology, 3724S). Secondary antibodies (all from Invitrogen) used were: anti-rabbit-HRP (32460), anti-mouse-HRP (32430), goat anti-rabbit Alexa Fluor 488 (A11034), goat anti-mouse Alexa Fluor 488 (A11029), goat anti-rabbit Alexa Fluor 594 (A11037) and goat anti-mouse Alexa Fluor 594 (A11032).

### Western blotting

Cell pellets were suspended in lysis buffer (20 mM Tris pH 8.0, 100 mM NaCl, 1 mM MgCl_2_, 0.1% IGEPAL) with fresh protease and phosphatase inhibitors added and incubated at 4°C for 15 min with rotation. Samples were then centrifuged at 13,000 rpm (15,871 ***g***) for 7 min. The supernatant was then collected and protein concentration measured by BCA assay (Pierce), and 30-50 µg of protein per well was run by SDS-PAGE and transferred to a nitrocellulose membrane (Cytiva Amersham) at 25 V overnight. All membranes were checked with Ponceau S staining for transfer efficiency and total protein loading. Membranes, except for CTC1 and TEN1 blots, were blocked with 1% non-fat milk in 1× PBS plus 0.1% Tween 20 (PBST) for at least 2 h. Membranes for CTC1 or TEN1 detection were blocked in 3% bovine serum albumin (BSA) in PBST. Primary antibodies were diluted in 1% non-fat milk-PBST or 3% BSA-PBST and incubated at 4°C overnight. Antibody dilutions were: STN1 1:2000, α-Actinin 1:10,000, Flag 1:1000, Cas9 1:2000, pATR T1989 1:1000, ATR 1:1000, pCHK1 1:CTC1 1:500, TEN1 1:1000, HA 1:1000. The membranes were then washed three times for 10 min each in PBST. Secondary antibodies were diluted in 1% non-fat milk-PBST or 3% BSA-PBST for at least 2 h at room temperature (RT). After incubation, the blots were then developed with Western Lightning Plus ECL (Perkin Elmer) or ECL Prime (Cytiva Amersham) and imaged on a Bio-Rad ChemiDoc MP or CL-XPosure film (Thermo Scientific). Uncropped images of western blots are shown in Fig. S5.

### IF

Cells were plated onto coverslips and allowed to grow for 24 h to 50-70% confluency. For all antibodies except phosphorylated histone H3 S10 (pH3 S10) in Fig. S1E, cells were pre-extracted with 0.1% Triton X-100 in CSK buffer (10 mM HEPES pH 7.4, 300 mM sucrose, 100 mM NaCl, 3 mM MgCl_2_) for 5 min at RT. Coverslips were then washed once with PBS and were fixed with 4% formaldehyde in PBS for 10 min at RT. After formaldehyde incubation, cells were rinsed once with PBS and then permeabilized with 0.5% Triton X-100 diluted in PBS for 10 min at RT. Following permeabilization, coverslips were washed with PBS and then stored 4°C in PBS until IF was performed. For the detection of pH3 Ser10 in Fig. S1E, the pre-extraction step was not performed. IF was then performed as previously described (Ackerson et al., 2020). Antibody dilutions were: RPA 1:500, γH2AX 1:1000, pH3 S10 1:500, pRPA32 S33 1:1000. For IF-FISH, IF was performed as described above and then telomere FISH was performed with a telomeric G-strand PNA probe (Alexa Fluor 488-[CCCTAA]_3_; PNA Bio), as previously described (Ackerson et al., 2020). Coverslips were mounted onto slides with Fluoromount-G containing 0.2 μg/ml DAPI. IF and IF-FISH images were taken on a Leica DMi8 fluorescence microscope. IF images for pH3 S10 in Fig. S1E and pH3 S10-RPA in Fig. S4 were taken under a 20× objective and the other IF images were taken under a 40× objective. The IF-FISH images were taken under a 63× objective. Foci and colocalizing foci were determined using ImageJ. DAPI images from the IF experiments were used to determine the number of micronuclei. At least five images and a minimum of 150 nuclei were analyzed per condition per independent, biological replicate for IF, except for the pH3 S10 and pH3 S10-RPA analyses (at least 50 nuclei were analyzed per independent, biological replicate). A minimum of 20 nuclei with three or more RPA-foci were analyzed per condition per independent, biological replicate for IF-FISH in Fig. 2C,D. Micronuclei were scored using DAPI images from RPA or RPA-γH2AX images for foci analysis in Figs 2 and 3.

### Southern blotting

Southern blot analysis was performed essentially as previously described (Kimura et al., 2010) with some modifications, as outlined below. Genomic DNA was isolated with the Promega Wizard Genomic DNA Purification Kit. DNA quality was checked, followed by digestion with RsaI and HinfI at 37°C overnight. The digested DNA was then run on an 0.8% 1× Tris-Acetate-EDTA (TAE) gel for 6 h at 50 V. The gel was depurinated, denatured and neutralized, as previously described, and the DNA transferred to a nylon membrane (Hybond N+, Amersham) by capillary action overnight. The membrane was pre-hybridized for at least 3 h at 60°C followed by hybridization with a 3′-digoxigenin (DIG)-labeled (CCCTAA)_3_ probe (Integrated DNA Technologies) at 60°C overnight. The membrane was washed, blocked, and incubated with an anti-DIG antibody (1:10,000) as described, except that the blocking and antibody incubation were for 1 h. The membrane was then washed and developed as previously described. The ladder probe was made with the DIG-High Prime DNA Labeling kit (Roche). Mean telomere length was calculated using the WALTER web-based toolset (Lycka et al., 2021).

### Anaphase bridges

Cells were plated on coverslips and allowed to grow overnight. Nocodazole was then added for 2 h, and cells were then washed three times with PBS and allowed to recover for 100-120 min. Cells were then fixed with 3% formaldehyde in PBS for 10 min. Coverslips were washed three times with PBS and mounted onto slides with Fluoromount-G containing 0.2 μg/ml DAPI. Fifty anaphase bridges were scored for each independent, biological replicate.

### Data analysis

At least three independent, biological replicates were completed for each experimental condition, as indicated in the figure legends. GraphPad Prism 10 software was used to calculate *P*-values. *P-*values were calculated using a two-tailed, unpaired *t*-test for all graphs, except for Fig. 2D, for which a Mann–Whitney *U*-test was used.

## Supplementary Material



10.1242/joces.264269_sup1 Supplementary information
