# Supplementary material for: Conditional deletion of human STN1 leads to telomere dysfunction, genome instability and proliferation defects
Source: J Cell Sci. 2026 May 18;139(10):jcs264269. doi: 10.1242/jcs.264269 (PMC13282564; doi:10.1242/jcs.264269)
Supplement: Supplementary information [file joces-139-264269-s1.pdf]

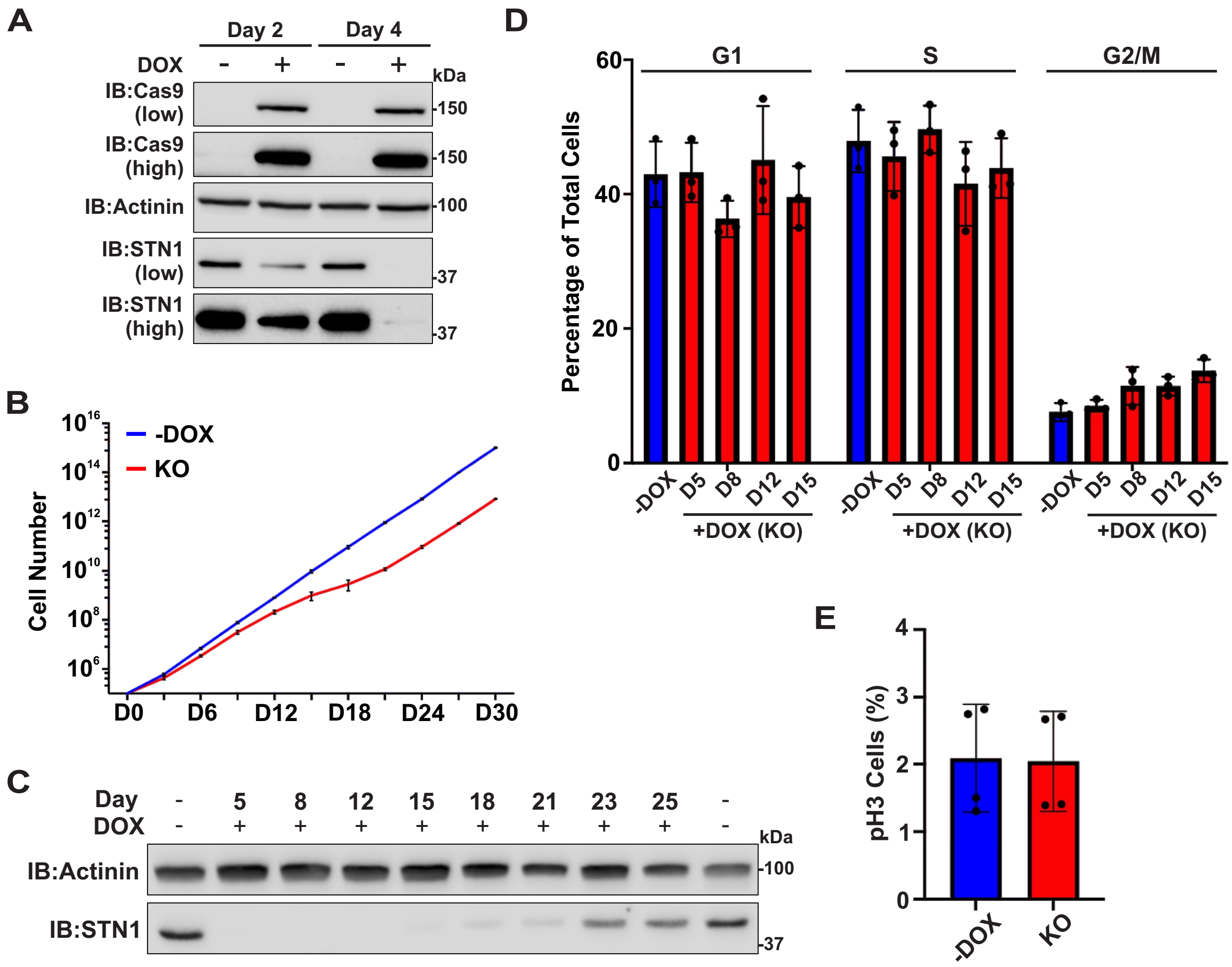

Fig. S1. Supporting material for Figure 1. (A) Western blot of Cas9 and STN1 levels in HeLa iCas9 sgSTN1 cells, as indicated. Actinin serves as a loading control. High and low indicate longer and shorter exposures of the blot, respectively. (B) Growth curve analysis of STN1 KO and control cells. D=day after DOX addition. (C) Western blot of STN1 levels over time after DOX addition. Actinin serves as a loading control. (D) Flow cytometry analysis of STN1 KO cells. Percentage of cells in different phases of the cell cycle. n=3 independent, biological replicates. (E) Percentage of mitotic cells as measured by the immunofluorescence of phosphorylated H3 S10 (pH3). n=4 independent, biological replicates. Average values in the graphs indicate the mean and error bars denote ±s.e.m. DOX=Doxycycline, D=day after DOX addition.

Journal of Cell Science • Supplementary information

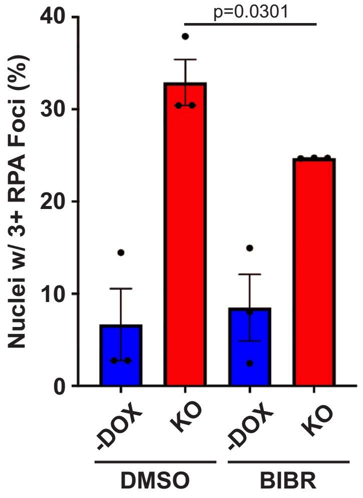

Fig. S2. Percentage of nuclei with 3 or more RPA foci in STN1 KO or control cells with or without BIBR treatment on day 15. n=3 independent, biological replicates. Average values in the graphs indicate the mean and error bars denote ±s.e.m. *P*-values were calculated by a two-tailed, unpaired *t*-test. DOX=Doxycycline.

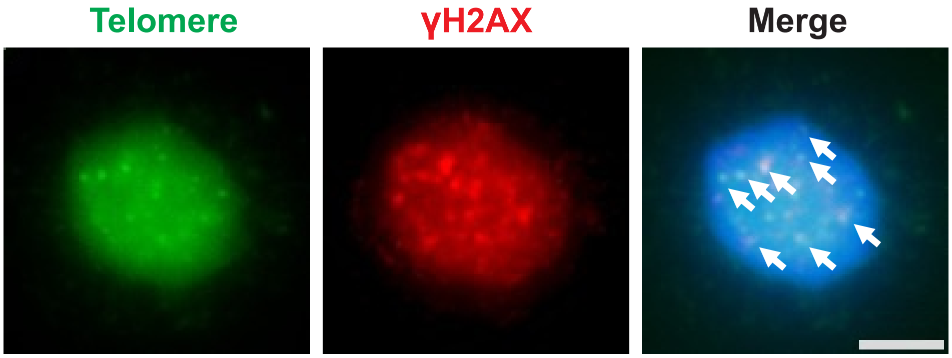

Fig. S3. Representative images of γH2AX localization to telomeres in STN1 KO cells on day 15 after DOX addition. The arrows denote co-localizations. Blue=DAPI staining. Scale bar=5 µm.

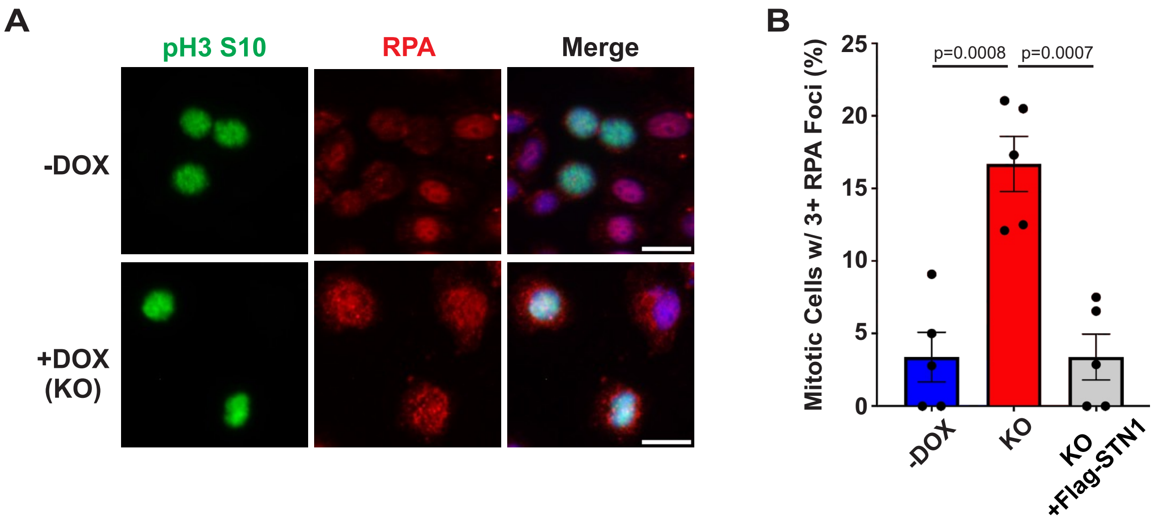

Fig. S4. Analysis of RPA foci in mitotic cells. (A) Representative images of pH3 S10-RPA foci. STN1 KO image is from day 15 after DOX addition. Blue=DAPI staining. Scale bar=20 μm (B) Analysis of foci in STN1 KO and control cells. Percentage of pH3 S10 positive nuclei displaying 3 or more RPA foci. KO +Flag-STN1 indicates STN1 KO cells expressing exogenous Flag-STN1. n=5 independent, biological replicates. Average values in the graphs indicate the mean and error bars denote ±s.e.m. *P*-values were calculated by a two-tailed, unpaired *t*-test. DOX=Doxycycline.

Journal of Cell Science • Supplementary information

Figure S5A

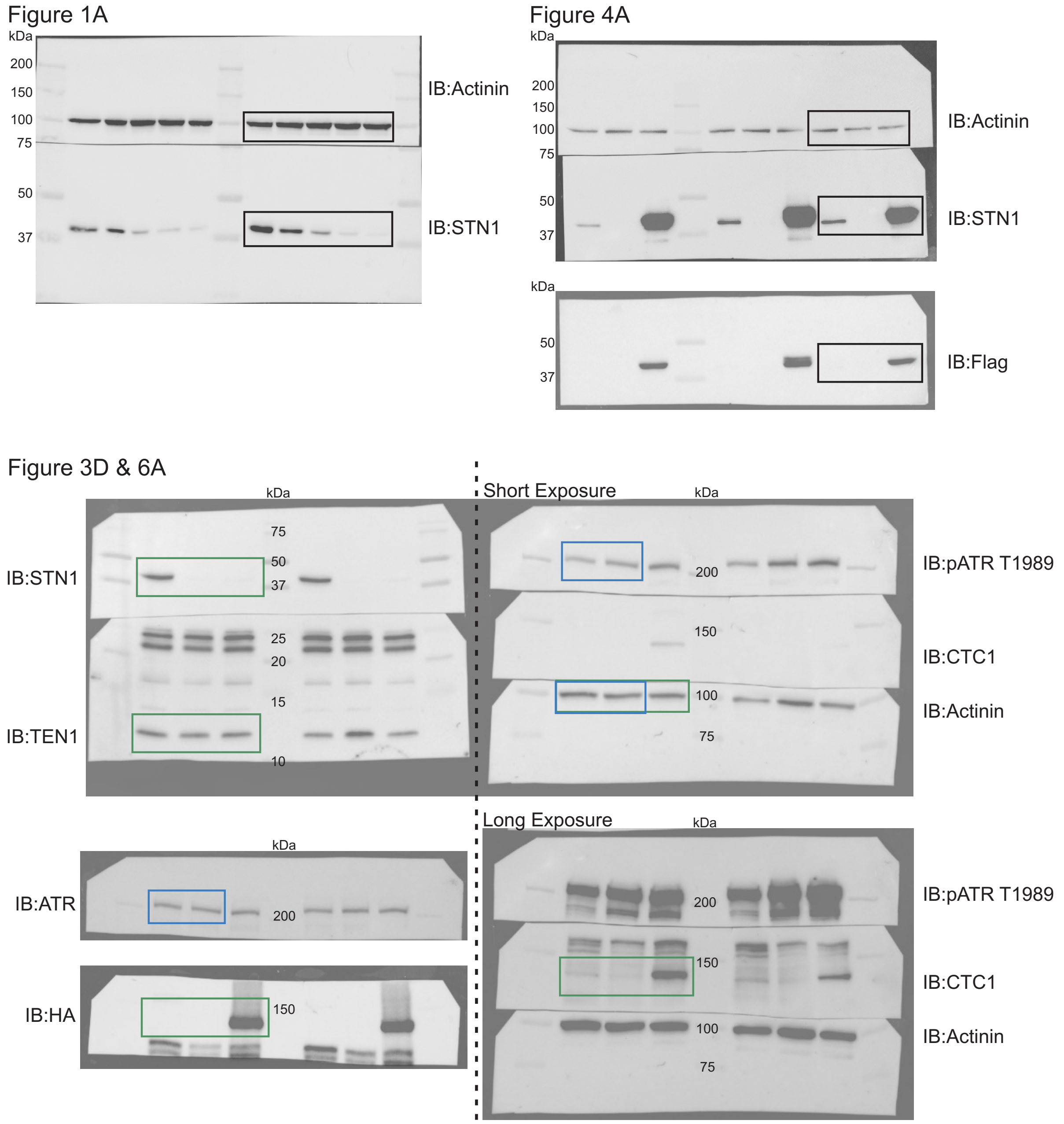

Journal of Cell Science • Supplementary information

Figure S5B

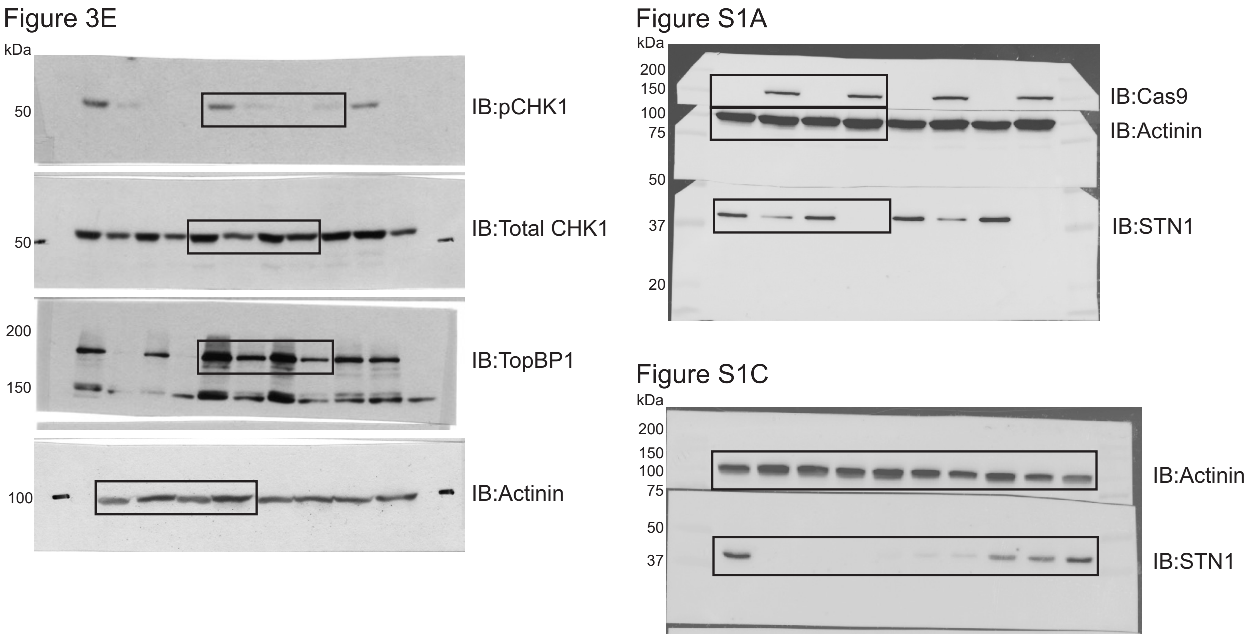

Fig. S5. Blot transparency. Full Western blots are shown for the corresponding figure, as indicated above the blots. Boxes identify regions that were included in the figures. For Fig. 3D (ATR), 4A (Flag), and 6A (HA), the original blot was stripped and re-probed with the indicated antibody. For Fig. 3D and 6A, the boxes in blue were used for Fig. 3D (pATR, ATR, Actinin) and the green boxes for Fig. 6A (CTC1, HA, STN1, TEN1, Actinin). All images, except Fig. 3E, were acquired on a gel imager, which includes the overlay of chemiluminescence/colorimetric images. The images for Fig. 3E were developed on X-ray film.

Journal of Cell Science • Supplementary information
