# Supplementary material for: Conditional deletion of human STN1 leads to telomere dysfunction, genome instability and proliferation defects
Source: J Cell Sci. 2026 May 18;139(10):jcs264269. doi: 10.1242/jcs.264269 (PMC13282564; doi:10.1242/jcs.264269)
Supplement: Supplementary file 1 [file joces-139-264269_review_history.pdf]

Journal of Cell Science

Conditional deletion of human STN1 leads to telomere dysfunction, genome instability and proliferation defects

Jaclyn S. Holbrooks, Colin A. Loveless, Kennedy A. Vogler, Steven Donte' Reed, Grayson H. Duvall, Carlan V. Romney, Stephanie M. Ackerson, Madison B. Kircher and Jason A. Stewart
DOI: 10.1242/jcs.264269

Editor: Renata Basto

Review timeline
Original submission: 3 July 2025
Editorial decision: 2 September 2025
First revision received: 18 March 2026
Accepted: 28 March 2026

Original submission

First decision letter

MS ID#: jcs.264269

MS TITLE: Conditional deletion of human STN1 leads to telomere dysfunction and telomerase-dependent genome instability and proliferation defects

AUTHORS: Jason Stewart; Jaclyn S Holbrooks; Colin A Loveless; Steven Donte' Reed; Grayson H Duvall; Carlan V Romney; Madison B Kircher

ARTICLE TYPE: Research Article

Dear Dr Stewart,

We have now reached a decision on the above manuscript.

To see the reviewers' reports and a copy of this decision letter, please go to: View Reviewer Comments

As you will see, the reviewers raise a number of substantial criticisms that prevent me from accepting the paper at this stage. They suggest, however, that a revised version might prove acceptable, if you can address their concerns. If you think that you can deal satisfactorily with the criticisms on revision, I would be pleased to see a revised manuscript.

Reviewer 1

Advance summary and potential significance to field

In this paper, the authors have set out to analyze the phenotypes of a STN1 Knock Out (KO) human cell line. STN1 is part of the conserved CST complex (that also comprises CTC1 and TEN1) that has several major functions in protecting the end of the linear chromosomes, called the telomeres. CST has important roles in the recruitment of telomerase and, consequently, on telomere length regulation and telomere end protection, and, as such, it is tightly involved in the loss of regulation of cell cycle control in tumor cells, as well as as in Coats Plus. There are important findings in the present study. However, the two principal findings are subject to caution and criticism, as explained below.

© 2026. Published by The Company of Biologists under the terms of the Creative Commons Attribution License (https://creativecommons.org/licenses/by/4.0/).

Comments for the author

General comments:
These major issues that need to be better documented before acceptance are:
1- The STN1 KO phenotype should be better documented. In particular, how can it be explained that the percentage of anaphase bridges (therefore in mitotic cells) become very abundant in STN1 KO cells at days 5-15 compared with the STN1 wt, while at the same time the percentage of H3 S10-P (a marker of mitosis) was unaffected in the KO cells compared to the control cells?
2- The effects of the telomerase inhibitor BIBR (which completely suppresses the STN1 KO arrest), albeit they might be very general, are interpreted by the authors as specifically due to the failure to generate the single-stranded G-OH extension. The conclusion that in the STN1 KO cells the inability of CST to inhibit telomerase results in G-OH overextension and telomeric damage provoking the arrest of these cells is highly speculative in the absence of additional experiments as proposed below.
For these reasons, I believe the data presented here are somewhat preliminary and that they could be better documented during revision concerning these two major issues. One is to better describe the STN1 KO phenotype with respect to the (yet not known accurately enough) relationship between the percentage of anaphase bridges and the position in the cell cycle (H3-S10-P). The second major issue, that concerning the nature of the damage supposed to activate the STN1 KO growth defects, might be addressed by performing additional experiments involving the relationships between the state of the G-OH extension and the STN1 KO phenotype. These additional experiments might involve the role of the DNA damage checkpoint in the detection of the G-overhang (as the authors had planned to do in a near future) as well as the possible effect of POT1 overexpression on the state of the G-overhang. Ideally, these experiments should be accompanied by measurements of the length of the G-overhang in each condition.
These experiments may appear too demanding, but it should be noted that once at least some of them can be incorporated in the current study, this will result in a very nice paper, much more convincing than in its present form. In their actual form, the present findings have a very limited impact on our general knowledge of the CST complex.

Major points:
1- On page 2 of the Introduction, it should be noted by the authors (perhaps just after the sentence of lines 23-25) that human (and mouse) OBFC1 was found to be the S. cerevisiae Stn1 homolog (based on sequences similarities) by the Ishikawa lab (Mol. Cell, 2009). These authors next performed mass spectrometry and identified CTC1 and TEN1 as co-precipitating with OBFC1/STN1. Then, these data allowed Casteel et al (2009) to understand that AAF-44 and AAF-132 previously identified by Goulian et al 1990 were in fact STN1 and CTC1, respectively. Therefore, the important role of the Ishikawa lab (Miyake et al 2009) in establishing the real nature of AAF-44 and AAF-132 should be acknowledged at this point of the manuscript, by stating that the homology with budding yeast Stn1 they uncovered was crucial. This point is particularly pertinent since the name « STN1 » for humans and mouse comes from the budding yeast nomenclature.
I acknowledge the fact that all these stories around CST discoveries are later described in the Discussion, page 8, lines 31-52. To me, these would be more pertinently placed in the Introduction, because they represent very general information of CST around the successive steps in the discovery of CST. On the other hand, more specific aspects of CST pathways as well as the analyses of CST KO lines are better suited to the Discussion as it has been done here in the original version.

2- In Figure 2, the authors found more RPA and gamma-H2AX in STN1 KO than in STN1 control cells. Next, the finding that the vast majority of RPA foci in KO cells is found at the telomeres is shown in Fig. S2. These data should be preferably shown as a main figure, as they represent an important finding, although it is true that, apparently, not many experiments were performed, which might weaken the conclusion. In addition, on line 61 of page 4, it is stated « To confirm that the RPA foci were telomeric… », but, if I understand correctly, this was not a confirmation because no data have shown that previously in the paper.
In addition, it is stated on line 55 of page 7 that the telomeric RPA foci occur at earlier time points than the growth defects. However, although the RPA foci do appear early and strongly around day 8, we cannot be certain that these are indeed telomeric foci, because the experiments in which RPA foci localize at the telomeres have not been apparently performed at different time points and, perhaps, not around day 8. Non telomeric RPA foci might appear early and be replaced later

© 2026. Published by The Company of Biologists under the terms of the Creative Commons Attribution License (https://creativecommons.org/licenses/by/4.0/).

by telomeric RPA foci. Given the importance of these data in the interpretation of STN1's roles, this point should be more accurately addressed.

3- In Fig. S1E the authors established that the percentage of mitotic cells (phosphorylated H3 S10) was the same in the KO cells as in the STN1 wt. Given this, then how can they explain that anaphase bridges (which are in mitotic cells and not in G2 cells) are much more abundant in STN1 KO cells than in the STN1 wt cells? Why were those STN1 KO cells with anaphase bridges (measured in Fig. 4) not previously detected as mitotic cells in the experiments of Fig. S1E?

4- The authors should be concerned by the fact that the telomerase inhibitor might not have for only consequence the failure to generate increased G-OH extensions. In other words, the effect of BIBR might not just affect the size of this G-overhang, but also have non specific effects on cell growth/proliferation. Indeed, just among other things, telomerase inhibition by BIBR provoke senescence resulting from the erosion of telomeres. Unfortunately, I do not see right now what the right control might be to eliminate this possibility. CTC1 KO cannot apparently serve as a negative control because it will be expected to behave like the STN1 KO in that matter.

5- In fact, the authors show that the inhibition of telomerase rescues almost completely the growth defects associated with STN1 KO but they have not proven that this effect was directly resulting from the state of the G-OH extension. A possible control could be to use POT1 overexpression in order to cure the defect due to too much G-overhangs. Overexpressed POT1 might significantly increase the occupancy of the G-OH extensions, thereby preventing RPA recruitment at these extensions and avoiding the STN1 KO growth defects.

6- In the Abstract, the statement that « these phenotypes and growth inhibition were largely dependent on telomerase activity » is not correct because inhibition of telomerase can lead to many different outcomes through indirect effects (not only on cell cycle progresssion) not necessarily linked to the activity of telomerase. I understand that this statement is dictated by the authors' hypothesis that the damage that causes STN1 KO growth defects originates from the generation of increased G-OH extension length that are aused by telomerase activity, but this just a hypothesis because activation of the DNA damage checkpoint was not measured in these experiments.

Minor points:
1- Throughout the manuscript, G-OH extension should be referred to as G-overhang or an approaching term (3' overhang, maybe?) that, to me, appears more widespread in the literature.

2- In Fig. 3, « dark » and « light » might not exactly be the appropriate terms, which perhaps would be better as « high exposure » and « low exposure » and, moreover, the low exposure is not very informative and might not be shown at all.

3- Scale bars are missing in several figures, for instance in Fig. 4, but also in other figures.

<u>Reviewer 2</u>

Advance summary and potential significance to field

This manuscript reports the consequences of the deletion of the STN1 subunit of the trimeric CTC1-STN1-TEN1 (CST) complex. The CST complex resembles the trimeric RPA complex and participates, among other things, to telomere replication, control of telomerase and C-strand fill-in.

Past studies have shown that:
Conditional deletion of CTC1 leads to reduced proliferation, accumulation of G2 cells, long, 3' telomeric overhangs and DDR signaling at telomeres (gH2AX). TEN1 deletion does not give the same phenotypes, but rather only a mild increase in telomeric overhang.

here the authors show that:

© 2026. Published by The Company of Biologists under the terms of the Creative Commons Attribution License (https://creativecommons.org/licenses/by/4.0/).

1) conditional deletion of STN1, resembles the CTC1 deletion phenotypes, i.e: reduced proliferation and accumulation of G2-cells. In addition, it confirms the accumulation of anaphase bridges and micronuclei previously-reported by the authors after knockdown of STN1.
2) Telomerase inhibition with BIBR1532 rescues the anaphase bridges and micronuclei.

The first result is somehow confirmatory, but also represents a useful set of experiments that complete the studies of the conditional alleles of this important complex. This part needs a couple of additional experiments to support the conclusions made.

The evidence that the RPA and gH2AX foci, induced after STN1 KO, are telomeric derives from one nucleus shown in figure S2, where telomeres and RPA are labelled by IF-FISH. No quantification is provided. To support their conclusion (RPA and gH2AX accumulation at telomeres) the authors should include in the main figures IF-FISH labeling of telomeres and RPA and/or telomeres and gH2AX, with quantification. This is important also given that we have no direct confirmation of the expected overhang phenotype here.

It would be useful to include an immunoblot for the other two subunits of the complex in Figure 1A, given that there is some degree of interdependency on the stability of the different subunits.

The second result, obtained with telomerase inhibition could be potentially interesting, but first the possibility that it is mediated by the effect of BIBR1532 on cell proliferation has to be ruled out. Both anaphase bridges and micronuclei increase over time as cells proliferate (Fig 4) These events are strictly related to cell division and the BIBR1532-treated KO cells divide significantly more slowly than the untreated KO cells (Fig 5E red vs magenta lines). Here I would also point out that the argument that BIBR1532 treatment almost completely rescues the proliferation of STN1-KO cells is not convincing. An "almost complete rescue" would mean growing more like the NT cells (blue line) while the BIBR1532-treated KO cells grow slower than all, even the BIBR1532-treated -Dox cells. All these sets of experiments are complicated by the effect of the BIBR1532-treatment on cell growth.
I wonder if this effect is even dependent on telomerease? Preventing telomere elongation would affect cell growth only after multiple passages that consume telomeres. Does this clone have short/very short telomeres?

Given the potential implication of this results, further controls (i.e. hTR/hTERT ko, or concentrations of BIBR that do not affect proliferation) are needed to support the conclusions.

Finally, the discussion is way too long and too broad, presenting almost as a review of the CST functions.

First revision

Author response to reviewers' comments

We thank the reviewers for their helpful comments and suggestions, and for taking the time to review the article. The suggested revisions have significantly improved the study and provided a more thorough characterization of STN1 KO. While addressed in more detail in specific comments to the reviewers' inquiries and concerns, below is a summary of the major revisions and additions to the manuscript:

1. Quantification of the RPA-telomere FISH analysis from D5-D15 across three trials has been added, which demonstrates an increase in telomeric RPA foci over time (Fig. 2C-D).

2. Telomere length analysis has been included across the time course (D5-D15) (Fig. 2E), following treatment with the telomerase inhibitor (BIBR1532) (Fig. 2F), and with add back of Flag-STN1 (Fig. 4C). These data demonstrate a telomerase-dependent elongation of some telomeres as well as a shortening of others, similar to what was observed in CTC1 KO

© 2026. Published by The Company of Biologists under the terms of the Creative Commons Attribution License (https://creativecommons.org/licenses/by/4.0/).

cells.

3. The telomeric and global DNA damage response have been further characterized with the addition of data showing increased phosphorylation of RPA32 S33 and ATR T1989 (ATR-specific modifications) (Fig. 3C-D) and decreased CHK1 activation following hydroxyurea treatment (Fig. 3E) in STN1 KO cells.

4. Analysis of RPA-foci in mitotic cells (pH3 S10) has been added, showing that cells with RPA foci can progress into mitosis (Fig. S4).

5. We attempted to address the comments from both reviewers, regarding the growth defect in cells treated with the telomerase inhibitor, by creating a STN1 KO cell line overexpressing POT1; however, the results were inconsistent with the telomerase inhibitor data. Additional experiments will be required to understand these differences. Accordingly, we have removed this data from the current study and adjusted the manuscript to reflect this change.

6. We now include analysis of CTC1 overexpression in the STN1 KO cells (Figure 6). During the review of this manuscript, a study was published, demonstrating that STN1 stabilizes CTC1 by preventing its degradation (Lan et al. Aging Cell. 2025). To test whether the phenotypes observed in the STN1 KO cells were solely due to the loss of CTC1, CTC1 was overexpressed in STN1 KO cells. Our data indicate that CTC1 overexpression is unable to rescue cell growth, telomere elongation, and genome instability following STN1 KO, indicating that STN1 also plays an essential role in telomere and genome maintenance outside of simply stabilizing CTC1.

7. The title, abstract, and manuscript have been revised and adjusted to reflect the new data added, the removal of the previous BIBR data, and the reviewers' concerns about the clarification of the general information on CST and discussion length. Textual changes to the manuscript are highlighted in blue in the "Revised Manuscript - Highlighted".

We hope that the new data and changes to the manuscript have adequately addressed the reviewers' concerns, improved our study, and that the manuscript is now suitable for publication. Comments to specific review comments are highlighted below.

Comments from the Reviewers:

Reviewer 1: SUMMARY OF THE ADVANCE MADE IN THIS PAPER AND ITS POTENTIAL SIGNIFICANCE TO THE FIELD In this paper, the authors have set out to analyze the phenotypes of a STN1 Knock Out (KO) human cell line. STN1 is part of the conserved CST complex (that also comprises CTC1 and TEN1) that has several major functions in protecting the end of the linear chromosomes, called the telomeres. CST has important roles in the recruitment of telomerase and, consequently, on telomere length regulation and telomere end protection, and, as such, it is tightly involved in the loss of regulation of cell cycle control in tumor cells, as well as as in Coats Plus. There are important findings in the present study. However, the two principal findings are subject to caution and criticism, as explained below.

SUGGESTIONS TO AUTHORS

General comments:

These major issues that need to be better documented before acceptance are:
1- The STN1 KO phenotype should be better documented. In particular, how can it be explained that the percentage of anaphase bridges (therefore in mitotic cells) become very abundant in STN1 KO cells at days 5-15 compared with the STN1 wt, while at the same time the percentage of H3 S10-P (a marker of mitosis) was unaffected in the KO cells compared to the control cells?

We have completed experiments to test for RPA foci in pH3 S10 positive cells (described in more

© 2026. Published by The Company of Biologists under the terms of the Creative Commons Attribution License (https://creativecommons.org/licenses/by/4.0/).

detail below). Moreover, we have included some additional characterization of the DNA damage response and telomere length analysis in the STN1 KO cells. Our previous work showed that CTC1 KO cells have pRPA32 S33 foci, increased pATR S1989, and are defective in CHK1 phosphorylation following HU treatment (Ackerson et al. Cell Cycle. 2020). We now include data demonstrating that STN1 KO cells also display these phenotypes (see Figure 3). Telomere length analysis is also included (see Figure 2).

2- The effects of the telomerase inhibitor BIBR (which completely suppresses the STN1 KO arrest), albeit they might be very general, are interpreted by the authors as specifically due to the failure to generate the single-stranded G-OH extension. The conclusion that in the STN1 KO cells the inability of CST to inhibit telomerase results in G-OH overextension and telomeric damage provoking the arrest of these cells is highly speculative in the absence of additional experiments as proposed below. For these reasons, I believe the data presented here are somewhat preliminary and that they could be better documented during revision concerning these two major issues. One is to better describe the STN1 KO phenotype with respect to the (yet not known accurately enough) relationship between the percentage of anaphase bridges and the position in the cell cycle (H3- S10-P). The second major issue, that concerning the nature of the damage supposed to activate the STN1 KO growth defects, might be addressed by performing additional experiments involving the relationships between the state of the G-OH extension and the STN1 KO phenotype. These additional experiments might involve the role of the DNA damage checkpoint in the detection of the G-overhang (as the authors had planned to do in a near future) as well as the possible effect of POT1 overexpression on the state of the G-overhang. Ideally, these experiments should be accompanied by measurements of the length of the G-overhang in each condition.

We understand the reviewer's concern regarding the growth defect caused by the telomerase inhibitor and how that may impact the interpretation of the data. Below, we describe our attempts to address this concern, which were not successful. As mentioned above, we have removed this data from the manuscript and revised it accordingly.

These experiments may appear too demanding, but it should be noted that once at least some of them can be incorporated in the current study, this will result in a very nice paper, much more convincing than in its present form. In their actual form, the present findings have a very limited impact on our general knowledge of the CST complex.

Major points:
1- On page 2 of the Introduction, it should be noted by the authors (perhaps just after the sentence of lines 23-25) that human (and mouse) OBFC1 was found to be the S. cerevisiae Stn1 homolog (based on sequences similarities) by the Ishikawa lab (Mol. Cell, 2009). These authors next performed mass spectrometry and identified CTC1 and TEN1 as co-precipitating with OBFC1/STN1. Then, these data allowed Casteel et al (2009) to understand that AAF-44 and AAF-132 previously identified by Goulian et al 1990 were in fact STN1 and CTC1, respectively. Therefore, the important role of the Ishikawa lab (Miyake et al 2009) in establishing the real nature of AAF-44 and AAF-132 should be acknowledged at this point of the manuscript, by stating that the homology with budding yeast Stn1 they uncovered was crucial. This point is particularly pertinent since the name « STN1 » for humans and mouse comes from the budding yeast nomenclature.

I acknowledge the fact that all these stories around CST discoveries are later described in the Discussion, page 8, lines 31-52. To me, these would be more pertinently placed in the Introduction, because they represent very general information of CST around the successive steps in the discovery of CST. On the other hand, more specific aspects of CST pathways as well as the analyses of CST KO lines are better suited to the Discussion as it has been done here in the original version.

We have moved this discussion to the introduction and included additional information about the history and nomenclature of the discovery of CST, as suggested (see Introduction, paragraphs 2-3).

2- In Figure 2, the authors found more RPA and gamma-H2AX in STN1 KO than in STN1 control cells. Next, the finding that the vast majority of RPA foci in KO cells is found at the telomeres is

© 2026. Published by The Company of Biologists under the terms of the Creative Commons Attribution License (https://creativecommons.org/licenses/by/4.0/).

shown in Fig. S2. These data should be preferably shown as a main figure, as they represent an important finding, although it is true that, apparently, not many experiments were performed, which might weaken the conclusion. In addition, on line 61 of page 4, it is stated « To confirm that the RPA foci were telomeric... », but, if I understand correctly, this was not a confirmation because no data have shown that previously in the paper.

In addition, it is stated on line 55 of page 7 that the telomeric RPA foci occur at earlier time points than the growth defects. However, although the RPA foci do appear early and strongly around day 8, we cannot be certain that these are indeed telomeric foci, because the experiments in which RPA foci localize at the telomeres have not been apparently performed at different time points and, perhaps, not around day 8. Non telomeric RPA foci might appear early and be replaced later by telomeric RPA foci. Given the importance of these data in the interpretation of STN1's roles, this point should be more accurately addressed.

We have changed the text to read "To determine whether RPA foci were telomeric,..." (page 2, paragraph 2, line 6).

We have also quantified the percentage of RPA co-localizing with telomeres between days 5 and 15 from three independent experiments (Figure 2C-D). It is interesting to note that RPA foci at days 5 and 8 are significantly increased above the -DOX control cells, but only a small portion of them are telomeric, suggesting that these foci may start from general genome instability and then become telomere-specific, as suggested by the reviewer. The results and discussion sections have been updated to reflect these new results.

3- In Fig. S1E the authors established that the percentage of mitotic cells (phosphorylated H3 S10) was the same in the KO cells as in the STN1 wt. Given this, then how can they explain that anaphase bridges (which are in mitotic cells and not in G2 cells) are much more abundant in STN1 KO cells than in the STN1 wt cells? Why were those STN1 KO cells with anaphase bridges (measured in Fig. 4) not previously detected as mitotic cells in the experiments of Fig. S1E?

We presumed that the STN1 KO cells must acquire a certain level of RPA-bound telomeres and DNA damage signaling, along with other associated genome instability, to arrest in G2. To test this idea, we determined the number of cells with 3+ RPA foci that are also pH3 S10 positive (Fig. S4). Our data suggest that some cells with RPA foci are not fully arrested in G2 and can still enter mitosis. We infer that either a certain threshold of RPA-bound telomeres must be present, or they are able to progress past the G2-arrest after a certain amount of time. We also note that the number of anaphase cells does not appear to differ between the STN1 KO and control cell lines, only the increase in anaphase bridges.

4- The authors should be concerned by the fact that the telomerase inhibitor might not have for only consequence the failure to generate increased G-OH extensions. In other words, the effect of BIBR might not just affect the size of this G-overhang, but also have non specific effects on cell growth/proliferation. Indeed, just among other things, telomerase inhibition by BIBR provoke senescence resulting from the erosion of telomeres. Unfortunately, I do not see right now what the right control might be to eliminate this possibility. CTC1 KO cannot apparently serve as a negative control because it will be expected to behave like the STN1 KO in that matter.

See below #5.

5- In fact, the authors show that the inhibition of telomerase rescues almost completely the growth defects associated with STN1 KO but they have not proven that this effect was directly resulting from the state of the G-OH extension. A possible control could be to use POT1 overexpression in order to cure the defect due to too much G-overhangs. Overexpressed POT1 might significantly increase the occupancy of the G-OH extensions, thereby preventing RPA recruitment at these extensions and avoiding the STN1 KO growth defects.

Since Reviewer 1, points #4 and #5 are related, we have responded to them together. To address the potential off-target effects of the telomerase inhibitor, we created a STN1 KO cell line that overexpressed POT1, similar to what was previously done in CTC1 KO cells (Fang et al. NAR. 2017). We predicted that this would rescue telomere damage signaling by preventing RPA binding.

© 2026. Published by The Company of Biologists under the terms of the Creative Commons Attribution License (https://creativecommons.org/licenses/by/4.0/).

However, while this cell line showed robust levels of Flag-POT1, the phenotypes (RPA foci, growth, cell cycle, anaphase bridges, and micronuclei) were not rescued. Why this does not rescue the phenotypes will require further characterization that extends beyond the scope of this work. Accordingly, we have removed the BIBR analysis (previous Figure 5) since we cannot rule out whether the growth defects and general genome instability are caused by other consequences of the telomerase inhibitor.

However, we have performed additional analysis of changes to telomere length and RPA binding to address G-overhang regulation following STN1 KO. After significant effort, we were able to measure telomere length in the STN1 KO cells by TRF Southern blotting. We now include TRF blots of telomere length from D5-D15 (Fig 2E), which shows both telomere elongation and telomere shortening. These changes correlate with increases in telomeric RPA seen by IF-FISH (Fig. 2C-D) and can be rescued with expression of Flag-STN1 in the KO cells (Fig. 4C). Additionally, we show that the elongated telomeres and RPA foci are telomerase-dependent (Fig. 2F & S2). Combined, these findings demonstrate that STN1 KO leads to G-overhang elongation and RPA binding, like what was observed in CTC1 KO cells (Feng et al. NAR. 2017; Feng et al. Nat Comm. 2018).

6- In the Abstract, the statement that « these phenotypes and growth inhibition were largely dependent on telomerase activity » is not correct because inhibition of telomerase can lead to many different outcomes through indirect effects (not only on cell cycle progresssion) not necessarily linked to the activity of telomerase. I understand that this statement is dictated by the authors' hypothesis that the damage that causes STN1 KO growth defects originates from the generation of increased G- OH extension length that are aused by telomerase activity, but this just a hypothesis because activation of the DNA damage checkpoint was not measured in these experiments.

The title, abstract, and manuscript have been updated to reflect the removal of the BIBR data and describe our new findings.

Minor points:
1- Throughout the manuscript, G-OH extension should be referred to as G-overhang or an approaching term (3' overhang, maybe?) that, to me, appears more widespread in the literature.

G-OH has been changed to G-overhang throughout the manuscript.

2- In Fig. 3, « dark » and « light » might not exactly be the appropriate terms, which perhaps would be better as « high exposure » and « low exposure » and, moreover, the low exposure is not very informative and might not be shown at all.

The changes have been made. The reason the low exposure is included is to demonstrate that the differences observed are not due to overexposure of the blot. Therefore, we prefer to keep them in the figures to provide proper representation and transparency of the band intensities.

3- Scale bars are missing in several figures, for instance in Fig. 4, but also in other figures.

Scale bars have been added.

Reviewer 2: This manuscript reports the consequences of the deletion of the STN1 subunit of the trimeric CTC1-STN1-TEN1 (CST) complex. The CST complex resembles the trimeric RPA complex and participates, among other things, to telomere replication, control of telomerase and C-strand fill-in.

Past studies have shown that:
Conditional deletion of CTC1 leads to reduced proliferation, accumulation of G2 cells, long, 3' telomeric overhangs and DDR signaling at telomeres (gH2AX). TEN1 deletion does not give the same phenotypes, but rather only a mild increase in telomeric overhang.

here the authors show that:

© 2026. Published by The Company of Biologists under the terms of the Creative Commons Attribution License (https://creativecommons.org/licenses/by/4.0/).

1) conditional deletion of STN1, resembles the CTC1 deletion phenotypes, i.e: reduced proliferation and accumulation of G2- cells. In addition, it confirms the accumulation of anaphase bridges and micronuclei previously-reported by the authors after knockdown of STN1.
2) Telomerase inhibition with BIBR1532 rescues the anaphase bridges and micronuclei.

The first result is somehow confirmatory, but also represents a useful set of experiments that complete the studies of the conditional alleles of this important complex. This part needs a couple of additional experiments to support the conclusions made.

The evidence that the RPA and gH2AX foci, induced after STN1 KO, are telomeric derives from one nucleus shown in figure S2, where telomeres and RPA are labelled by IF-FISH. No quantification is provided. To support their conclusion (RPA and gH2AX accumulation at telomeres) the authors should include in the main figures IF-FISH labeling of telomeres and RPA and/or telomeres and gH2AX, with quantification. This is important also given that we have no direct confirmation of the expected overhang phenotype here.

Reviewer 1 also shared this concern, and we have added this data. Please see our response to Reviewer 1, point #2 and Figure 2C-D.

It would be useful to include an immunoblot for the other two subunits of the complex in Figure 1A, given that there is some degree of interdependency on the stability of the different subunits.

A blot with both CTC1 and TEN1 levels in the STN1 KO has now been included (Figure 6A). As outlined in the summary above, CTC1 levels are reduced following STN1 KO. We have addressed whether the loss of CTC1 is causing the phenotypes in the STN1 KO cells by overexpressing an HA-tagged CTC1 (Figure 6). TEN1 levels showed little to no change in the STN1 KO cells.

The second result, obtained with telomerase inhibition could be potentially interesting, but first the possibility that it is mediated by the effect of BIBR1532 on cell proliferation has to be ruled out. Both anaphase bridges and micronuclei increase over time as cells proliferate (Fig 4) These events are strictly related to cell division and the BIBR1532-treated KO cells divide significantly more slowly than the untreated KO cells (Fig 5E red vs magenta lines). Here I would also point out that the argument that BIBR1532 treatment almost completely rescues the proliferation of STN1-KO cells is not convincing. An "almost complete rescue" would mean growing more like the NT cells (blue line) while the BIBR1532-treated KO cells grow slower than all, even the BIBR1532-treated -Dox cells. All these sets of experiments are complicated by the effect of the BIBR1532- treatment on cell growth.

I wonder if this effect is even dependent on telomerease? Preventing telomere elongation would affect cell growth only after multiple passages that consume telomeres. Does this clone have short/very short telomeres?

Given the potential implication of this results, further controls (i.e. hTR/hTERT ko, or concentrations of BIBR that do not affect proliferation) are needed to support the conclusions.

Reviewer 1 also shared these concerns. Please see our response to Reviewer 1, major points #4-6.

Finally, the discussion is way too long and too broad, presenting almost as a review of the CST functions.

The discussion of CST discovery and functions has been shortened and moved to the introduction, as suggested by Reviewer
1. Additionally, we removed the discussion subheading “Potential implications for understanding CST-associated telomere biology disorders” since this section was mostly related to the telomerase inhibitor experiments.

© 2026. Published by The Company of Biologists under the terms of the Creative Commons Attribution License (https://creativecommons.org/licenses/by/4.0/).

Second decision letter

MS ID#: jcs.264269R1

MS Title: Conditional deletion of human STN1 leads to telomere dysfunction, genome instability, and proliferation defects

Authors: Jaclyn S Holbrooks; Colin A Loveless; Kennedy A Vogler; Steven Donte' Reed; Grayson H Duvall; Carlan V Romney; Stephanie M Ackerson; Madison B Kircher; Jason Stewart

Article Type: Research Article

Dear Dr Stewart,

I am happy to tell you that your manuscript has been accepted for publication in Journal of Cell Science, pending standard publication integrity checks.

© 2026. Published by The Company of Biologists under the terms of the Creative Commons Attribution License (https://creativecommons.org/licenses/by/4.0/).
